# Sedation for awake tracheal intubation: A systematic review and network meta‐analysis

**DOI:** 10.1111/anae.16452

**Published:** 2024-10-28

**Authors:** Kariem El‐Boghdadly, Neel Desai, Jordan B. Jones, Sally Elghazali, Imran Ahmad, J. Robert Sneyd

**Affiliations:** ^1^ Department of Anaesthesia Guy's and St Thomas' NHS Foundation Trust London UK; ^2^ King's College London London UK; ^3^ University of Florida College of Medicine Gainesville FL USA; ^4^ Department of Anaesthesia Northwick Park Hospital London UK; ^5^ Peninsula Medical School University of Plymouth Plymouth UK

**Keywords:** airway, awake intubation, drugs, network meta‐analysis, sedation

## Abstract

**Background:**

Different sedation regimens have been used to facilitate awake tracheal intubation, but the evidence has not been synthesised robustly, particularly with respect to clinically important outcomes. We conducted a systematic review and network meta‐analysis to determine the sedation techniques most likely to be associated with successful tracheal intubation, a shorter time to successful intubation and a lower risk of arterial oxygen desaturation.

**Methods:**

We searched for randomised controlled trials of patients undergoing awake tracheal intubation for any indication and reporting: overall tracheal intubation success rate; tracheal intubation time; incidence of arterial oxygen desaturation; and other related outcomes. We performed a frequentist network meta‐analysis for these outcomes if two or more sedation regimens were compared between included trials. We also performed a sensitivity analysis excluding trials with a high risk of bias.

**Results:**

In total, 48 studies with 2837 patients comparing 33 different regimens were included. Comparing overall awake tracheal intubation success rates (38 studies, 2139 patients), there was no evidence suggesting that any individual sedation regimen was superior. Comparing times to successful tracheal intubation (1745 patients, 24 studies), any sedation strategy was superior to placebo. When we excluded trials with a high risk of bias, we found no evidence of a difference between any interventions for time to successful tracheal intubation. Thirty‐one studies (1753 patients) suggested that dexmedetomidine and magnesium sulphate were associated with a reduced risk of arterial oxygen desaturation compared with other interventions, but excluding trials with a high risk of bias suggested no relevant differences between interventions. The quality of evidence for each of our outcomes was low.

**Conclusions:**

To maximise effective and safe awake tracheal intubation, optimising oxygenation, topical airway anaesthesia and procedural performance may have more impact than any given sedation regimen.

## Introduction

Patients with an anticipated difficult airway are at risk of complications from airway management under general anaesthesia. The Difficult Airway Society (DAS) recommends that awake tracheal intubation (ATI) should be considered for these patients [1]. The key principles of ATI include oxygenation; topical anaesthesia of the airway; procedural performance; and sedation. While sedation is not essential for ATI [2], cautious use of minimal sedation can reduce anxiety and discomfort, provide amnesia and increase procedural tolerance [3]. However, adverse consequences of procedural sedation include hypoventilation; airway obstruction; oxygen desaturation; and cardiovascular instability. Therefore, appropriate selection of drug regimens is crucial to facilitate safe and effective ATI.

A range of sedation strategies have been used in an effort to enhance safety while providing sufficient efficacy. Clinicians remain uncertain as to the ideal drugs to utilise, and DAS guidelines highlight that a range of strategies can be used [1]. Several studies have attempted to synthesise existing evidence, but there have been significant limitations to each. Some only provide qualitative synthesis, and thus objective assessment of relevant outcomes is difficult for clinicians to interpret [3, 4]. Other studies only assess one or two individual drugs, ignoring the heterogeneity in available drugs and combinations [5, 6]. A study by Cabrini et al. interpreted sedation strategies in conjunction with techniques for topical anaesthesia, and conducted pairwise meta‐analyses with modest numbers of studies, suggesting benefits to dexmedetomidine [7]. Importantly, outcomes that might have an impact on patient experience have not been prioritised, such as tracheal intubation success rate and time to successful tracheal intubation. There is, therefore, a need to quantitatively synthesise the evidence base in light of this heterogeneity.

We conducted a systematic review and network meta‐analysis with the aims of determining the sedation techniques most likely to be associated with successful ATI, with the shortest time to perform, in the absence of adverse outcomes.

## Methods

This systematic review and network meta‐analysis was conducted and reported in accordance with PRISMA‐NMA recommendations [8].

We searched for randomised controlled trials of adult patients in any clinical setting undergoing ATI for any indication. The main outcomes were: overall tracheal intubation success rate; time to tracheal intubation; and incidence of arterial oxygen desaturation (as defined within each individual study). Other outcomes included need for rescue sedation (additional sedation not forming the primary sedative technique); time to conduct fibrescopy; first attempt tracheal intubation success rate; and incidence of adverse cardiovascular events (bradycardia, tachycardia, hypotension, hypertension and arrhythmia). Awake tracheal intubations via nasal or oral route were included, and oxygenation, topical anaesthesia or procedural performance strategies did not affect inclusion. Studies not published in English or not reporting any of these outcomes were not included.

We searched Embase, MEDLINE, PubMed and Cochrane CENTRAL databases and supplemented this by hand searching reference lists of included studies. Databases were searched from inception through to 10 November 2021, with the search updated on 23 September 2023. Search terms relating to ATI, tracheal intubation and sedation were used in various permutations and combined with Boolean operators (online Supporting Information Appendix S1). Articles were de‐duplicated using Mendeley Desktop (Elsevier, Amsterdam, Netherlands), then imported for title, abstract and full‐text screening according to our eligibility criteria by two authors (SE, JJ) independently into Rayyan software [9]. In the case of uncertainty, a third reviewer (KE) adjudicated.

Data were extracted by two authors (SE, JJ) onto a standardised Microsoft Excel spreadsheet (Microsoft, Inc., Redmond, WA, USA). Two other authors (ND, KE) validated the extracted data. Data collected included study demographics; patient characteristics; indication for awake intubation; methods of sedation in clinical area where ATI was performed; sedation team members; experience of airway operators; and outcomes. Risk of bias assessment was performed by two authors (SE, JJ) and adjudicated by a third (KE), using the Cochrane risk of bias (RoB) 2 tool [10]. Data were then transcribed from Microsoft Excel into Stata (Version 16.1, StataCorp LLC, College Station, TX, USA) by one author and crosschecked by a second author.

We planned to conduct a network meta‐analysis using a frequentist statistical method if more than two different sedation regimens for a particular outcome of interest could be linked into a network through direct comparisons between the included trials [11, 12]. A network plot was then produced for each outcome subjected to network meta‐analysis that shared a common heterogeneity parameter and multivariate model. The nodes depicted the interventions and the connecting lines represented the direct comparisons between these interventions. The size of the nodes and width of the lines were related to the relative quantity of data involved. Summary mean (SD) data of a particular outcome across all trials were provided. Indirect comparisons were mathematically derived from direct comparison estimates with a common comparator. Importantly, the assumption of coherence between direct and indirect estimates was confirmed statistically using the local separating indirect from direct evidence technique and global design by treatment interaction test. Network meta‐analysis was not conducted if the assumption of coherence between direct and indirect estimates was not confirmed statistically. The results of direct and indirect comparisons between interventions were aggregated into network league tables. These were presented as mean differences or odds ratios with their confidence intervals for continuous and dichotomous outcomes, respectively. Sensitivity analyses were performed for our three main outcomes but excluding trials at high risk of bias. Interventions were then ranked in the absence of serious imprecision for each of our outcomes.

The quality of evidence for outcomes was assessed by two authors (ND, KE) with respect to the GRADE system using Confidence in Network Meta‐Anlaysis (CINeMA) software (Institute of Social and Preventative Medicine, University of Bern, Switzerland) [13]. Five individual domains, namely: limitations; indirectness; imprecision; inconsistency; and publication bias, were evaluated for seriousness. The minimal clinically important differences for precision were set by agreement among the authors based on their clinical judgement at 5% for overall tracheal intubation success rate; 60 s for time to tracheal intubation; 10% for incidence of arterial oxygen desaturation; 5% for need for rescue sedation; 60 s for time to conduct fibrescopy; 5% for first attempt tracheal intubation success rate; and 10% for incidence of arterial oxygen desaturation. We assessed publication bias with a comparison‐adjusted funnel plot and Egger's linear regression test. If seriousness was present in any of these domains, the quality of evidence was then downgraded.

## Results

**Figure 1:**
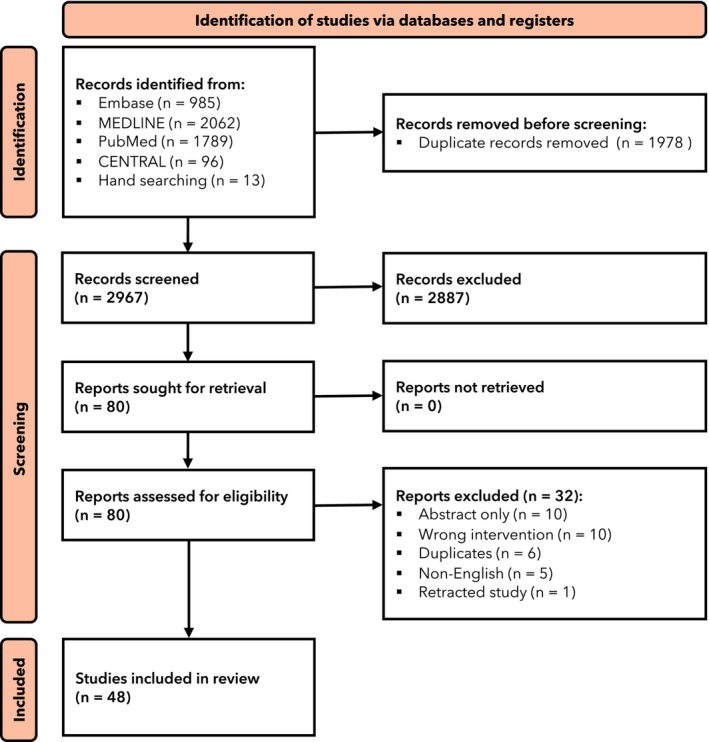
Study flow diagram summarising the retrieved, included and the excluded randomised controlled trials.

Following screening (Fig. 1), 48 trials with 2837 patients were included in the systematic review [14, 15, 16, 17, 18, 19, 20, 21, 22, 23, 24, 25, 26, 27, 28, 29, 30, 31, 32, 33, 34, 35, 36, 37, 38, 39, 40, 41, 42, 43, 44, 45, 46, 47, 48, 49, 50, 51, 52, 53, 54, 55, 56, 57, 58, 59, 60, 61]. The following drugs were examined: alfentanil [25]; dexmedetomidine with fentanyl [55]; diazepam [42]; diazepam with alfentanil [42]; fentanyl with ketamine [50]; magnesium sulphate [21]; midazolam with clonidine [37]; midazolam with fentanyl with ketamine [18]; midazolam with fentanyl with propofol [18]; midazolam with fentanyl with remifentanil [18]; midazolam with remifentanil [40]; midazolam with sufentanil [31]; nalbuphine [60]; perineural dexmedetomidine with propofol [35]; remifentanil with propofol [14] and sufentanil [45] in one trial each; fentanyl with propofol [28, 47]; ketamine with propofol [19, 22] and midazolam with propofol [33, 40] in two trials; dexmedetomidine with ketamine [22, 27, 46] in three trials; dexmedetomidine with propofol [19, 23, 27, 35]; midazolam with dexmedetomidine [31, 37, 52, 57] and placebo [21, 38, 48, 61] in four trials; fentanyl [16, 26, 36, 41, 60] and midazolam [21, 34, 37, 44, 57] in five trials; midazolam with fentanyl [15, 28, 33, 39, 43, 52, 53, 56] and propofol [17, 23, 29, 30, 35, 49, 54, 59] in eight trials; remifentanil in 10 trials [20, 24, 29, 32, 39, 51, 54, 58]; and dexmedetomidine [16, 17, 24, 25, 26, 32, 33, 34, 38, 41, 43, 45, 46, 47, 48, 49, 50, 51, 53, 55, 56, 61] in 28 trials [15, 16, 17, 20, 24, 25, 26, 32, 33, 34, 36, 38, 41, 43, 44, 45, 46, 47, 48, 49, 50, 51, 53, 55, 56, 58, 59, 61].

The results of the risk of bias assessment are shown in online Supporting Figure S1. The overall risk of bias was low in 24 trials [16, 18, 19, 24, 25, 31, 32, 33, 34, 35, 36, 37, 38, 43, 44, 45, 46, 47, 48, 49, 51, 52, 60, 61], there were some concerns in 10 [17, 21, 26, 40, 42, 50, 54, 55, 56, 57] and was high in 14 trials [14, 15, 20, 22, 23, 27, 28, 29, 30, 39, 41, 53, 58, 59]. Seven of the 35 authors who were emailed for further information responded [18, 19, 29, 33, 46, 59, 60].

Characteristics of included trials are presented in online Supporting Information Table S1. The sedation provider and airway operator were different people, the same person and not specified, respectively, in 27 [14, 15, 16, 19, 21, 23, 24, 25, 26, 31, 32, 33, 35, 36, 37, 40, 43, 44, 46, 47, 49, 51, 54, 56, 58, 60, 61], four [18, 29, 52, 59] and 17 [17, 20, 22, 27, 28, 30, 34, 38, 39, 41, 42, 45, 48, 50, 53, 55, 57] trials, respectively. The airway operator was a trainee in two trials [32, 58]; consultant, experienced, senior and/or expert in 28 trials [14, 16, 18, 19, 21, 23, 24, 25, 26, 29, 30, 31, 33, 35, 36, 37, 38, 40, 43, 44, 46, 47, 49, 51, 52, 54, 59, 61]; and not specified in 18 trials [15, 17, 20, 22, 27, 28, 34, 39, 41, 42, 45, 48, 50, 53, 55, 56, 57, 60]. Topical anaesthesia to facilitate ATI was used in all studies but one [30] with a range of strategies utilised (online Supporting Information Table S1).

**Figure 2:**
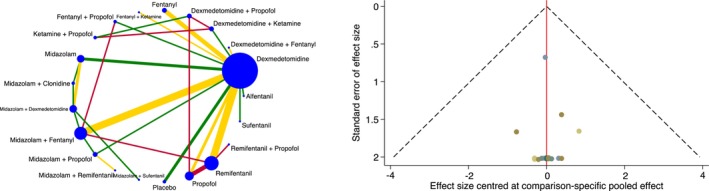
Network plot for overall ATI success rate (left). Each intervention is depicted by a circle that is proportional in size to the number of patients who were randomised to that intervention. Connecting lines between the circles indicate the direct comparisons of interventions, their width proportional to the number of trials evaluating the comparison and their colour representing the average risk of bias. Green, low risk; yellow, some concerns; and red, high risk. Comparison‐adjusted funnel plot for overall ATI success rate (right). Different colours correspond to particular comparisons of interventions. The red line indicates the null hypothesis that the comparison‐specific pooled effect estimates do not differ from the respective trial‐specific effect sizes.

Overall tracheal intubation success rate was investigated in 2246 patients and 40 trials [14, 15, 17, 19, 20, 22, 23, 24, 25, 26, 27, 28, 29, 30, 31, 32, 33, 34, 36, 37, 38, 39, 40, 41, 43, 44, 45, 46, 47, 48, 49, 50, 51, 52, 53, 54, 55, 56, 57, 58], with an overall mean (SD) success rate of 99.3% (2.2%). In the network plot, 28 direct and 182 indirect comparisons were established between 21 interventions (Fig. 2). No significant differences were shown between different sedation strategies and overall tracheal intubation success rate (online Supporting Information Table S2). After excluding trials with a high risk of bias, no changes in the significance of results were observed (online Supporting Information Appendix S2). The standard deviation of the between‐trial heterogeneity was 5.33 × e^‐9^. Local and global inconsistency were absent. Publication bias was not found, neither on inspection of the comparison‐adjusted funnel plot nor on performance of Egger's test (p = 0.70) (Fig. 2). Given the presence of serious limitations and imprecision, the quality of evidence for this outcome was rated low (Table 1 and online Supporting Information Appendix S3).

**Table 1:** Summary of conclusions from the results of the network meta‐analysis and GRADE quality of evidence evaluation. Full table is available in online Supporting Information Table S3. Values are number and mean (SD).

Outcome	Patients /interventions (n)	Direct /indirect comparisons (n)	Outcome measure	Quality of evidence	Comments
Overall awake tracheal intubation success rate; % [14, 15, 17, 19, 20, 22, 23, 24, 25, 26, 27, 28, 29, 30, 31, 32, 33, 34, 36, 37, 38, 39, 40, 41, 43, 44, 45, 46, 47, 48, 49, 50, 51, 52, 53, 54, 55, 56, 57, 58]	2246/21	28/182	99% (2.2%)	Low (⊕⊕)	No local or global inconsistency Downgraded for serious limitations and imprecision
Time to tracheal intubation; s [16, 17, 19, 20, 21, 22, 23, 24, 25, 26, 31, 32, 33, 37, 39, 40, 43, 45, 47, 49, 50, 51, 52, 56]	1745 /20	23/167	237 s (319 s)	Low (⊕⊕)	No local or global inconsistency Downgraded for serious limitations and imprecision
Incidence of arterial oxygen desaturation; % [16, 17, 19, 20, 21, 23, 24, 25, 27, 29, 30, 31, 32, 33, 34, 36, 38, 39, 40, 41, 43, 45, 46, 47, 49, 50, 51, 52, 53, 54, 58, 60]	1813/20	25/165	10% (18.5%)	Low (⊕⊕)	No local or global inconsistency Downgraded for serious limitations and imprecision
Need for rescue sedation; % [24, 29, 32, 33, 43, 47, 48, 54, 61]	578/7	7/14	26% (31.9%)	Low (⊕⊕)	No local or global inconsistency Downgraded for serious imprecision and publication bias
Time to conduct fibrescopy (s) [24, 25, 33, 40, 43, 50, 56]	351/7	7/14	132 (56.4)	Low (⊕⊕)	No local or global inconsistency Downgraded for serious limitations and imprecision
Incidence of adverse cardiovascular events (%) [14, 16, 17, 20, 23, 24, 25, 29, 30, 31, 34, 37, 38, 41, 45, 46, 47, 48, 49, 50, 51, 52, 53, 55, 58]	1323/18	20/133	21 (25.8)	Low (⊕⊕)	No local or global inconsistency Downgraded for serious limitations and imprecision

**Figure 3:**
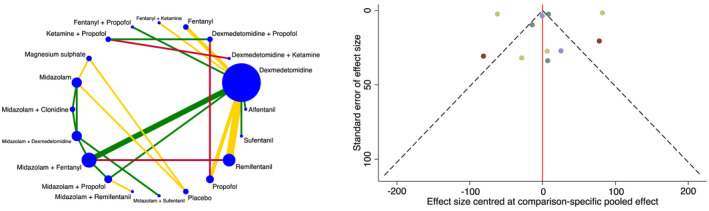
Network plot for time to tracheal intubation (left). Each intervention is depicted by a circle that is proportional in size to the number of patients who were randomised to that intervention. Connecting lines between the circles indicate the direct comparisons of interventions, their width proportional to the number of trials evaluating the comparison and their colour representing the average risk of bias. Green, low risk; yellow, some concerns; and red, high risk. Comparison‐adjusted funnel plot for time to tracheal intubation (right). Different colours correspond to particular comparisons of interventions. The red line indicates the null hypothesis that the comparison‐specific pooled effect estimates do not differ from the respective trial‐specific effect sizes.

Time to tracheal intubation was investigated in 1745 patients and 24 trials [16, 17, 19, 20, 21, 22, 23, 24, 25, 26, 31, 32, 33, 37, 39, 40, 43, 45, 47, 49, 50, 51, 52, 56], with an overall mean (SD) of 237 (319) s. In the network plot, 23 direct and 167 indirect comparisons were made between 20 interventions (Fig. 3). Placebo was inferior to all other interventions. Dexmedetomidine was superior to fentanyl. Dexmedetomidine with ketamine and ketamine with propofol decreased the time to tracheal intubation compared with: alfentanil; dexmedetomidine; dexmedetomidine with propofol; fentanyl; fentanyl with ketamine; fentanyl with propofol; midazolam; midazolam with clonidine; midazolam with dexmedetomidine; midazolam with fentanyl; midazolam with propofol; midazolam with remifentanil; midazolam with sufentanil; propofol; remifentanil; and sufentanil.

Furthermore, magnesium sulphate was superior to: alfentanil; dexmedetomidine; dexmedetomidine with propofol; fentanyl; fentanyl with ketamine; midazolam; midazolam with clonidine; midazolam with dexmedetomidine; midazolam with fentanyl; midazolam with propofol; and propofol. Midazolam with dexmedetomidine was superior to midazolam. Remifentanil reduced the time to tracheal intubation relative to dexmedetomidine; fentanyl; and midazolam with fentanyl. No other statistical differences were shown between interventions.

Sensitivity analysis excluding trials with a high risk of bias showed that dexmedetomidine was no longer superior to fentanyl; dexmedetomidine with ketamine and ketamine with propofol did not form part of the network league table; magnesium sulphate was not superior to alfentanil, fentanyl with ketamine and propofol; midazolam with dexmedetomidine was not superior to midazolam; and remifentanil was no longer superior to dexmedetomidine and midazolam with fentanyl.

The standard deviation of the between‐trial heterogeneity was 50.2. Local and global inconsistency were absent. Publication bias was not found, neither on inspection of the comparison‐adjusted funnel plot nor on performance of Egger's test (p = 0.80) (Fig. 3). In view of the presence of serious limitations and imprecision, the overall quality of evidence for time to tracheal intubation was graded as low.

**Figure 4:**
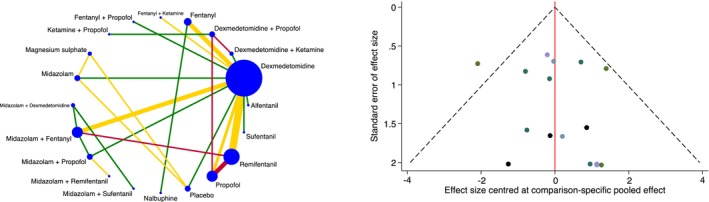
Network plot for incidence of arterial oxygen desaturation (left). Each intervention is depicted by a circle that is proportional in size to the number of patients who were randomised to that intervention. Connecting lines between the circles indicate the direct comparisons of interventions, their width proportional to the number of trials evaluating the comparison and their colour representing the average risk of bias. Green, low risk; yellow, some concerns; and red, high risk. Comparison‐adjusted funnel plot for incidence of arterial oxygen desaturation (right). Different colours correspond to particular comparisons of interventions. The red line indicates the null hypothesis that the comparison‐specific pooled effect estimates do not differ from the respective trial‐specific effect sizes.

The incidence of arterial oxygen desaturation was reported in 1813 patients and 32 trials [16, 17, 19, 20, 21, 23, 24, 25, 27, 29, 30, 31, 32, 33, 34, 36, 38, 39, 40, 41, 43, 45, 46, 47, 49, 50, 51, 52, 53, 54, 58, 60], with an overall mean (SD) of 9.7% (18.5%). In the network plot, 25 direct and 165 indirect comparisons were made between 20 interventions (Fig. 4). Dexmedetomidine reduced the incidence of oxygen desaturation compared with fentanyl and propofol. Moreover, magnesium sulphate was superior to: fentanyl; midazolam; midazolam with fentanyl; midazolam with propofol; midazolam with remifentanil; placebo; propofol; and remifentanil. No other statistical differences were shown between interventions. After excluding trials with a high risk of bias, magnesium was no longer superior to midazolam with fentanyl and midazolam with remifentanil but it was now superior to nalbuphine. Fentanyl was also now inferior to midazolam, remifentanil and sufentanil. The standard deviation of the between‐trial heterogeneity was 0.75. Local and global inconsistency were absent. Publication bias was not found, neither on inspection of the comparison‐adjusted funnel plot nor on performance of Egger's test (p = 0.12) (Fig. 4). Given the presence of serious limitations and imprecision, the overall quality of evidence for the incidence of arterial oxygen desaturation was graded as low.

Details of the results of the other outcomes are presented in Table 1 (see also online Supporting Information Table S3 and Appendix S4). Given the presence of global inconsistency for the first attempt tracheal intubation success rate, network meta‐analysis was not conducted for this outcome. To avoid disconnection of the network plot and facilitate statistical analysis, some trials had to be excluded from the network meta‐analysis, and the results of these trials have been reported in online Supporting Information Table S4 [18, 19, 40, 42, 59].

## Discussion

This systematic review and network meta‐analysis found a wide variety of sedation regimens that were delivered most frequently by independent sedation providers while senior anaesthetists performed tracheal intubation. Although the quality of evidence was generally low, despite a large number of patients included, we showed that successful ATI does not appear related to sedation strategy. Moreover, we found that dexmedetomidine either as a sole drug, or combined with ketamine or propofol, was most likely to facilitate timely ATI, although this was no longer apparent when trials with a high risk of bias were excluded. Notably, and contrary to previous data, remifentanil was not shown to be superior to any other intervention for our outcomes of interest. However, although sedation regimens may have been favourable for different outcomes, there was no one individual regimen that was clearly better over a range of relevant outcomes.

Our first outcome is important, as successful ATI means patients have their airways managed safely and can then proceed to surgery following induction of general anaesthesia. Success or failure of airway management is a fundamental determinant of peri‐operative care and thus patient outcomes. It is notable, therefore, that the overall success rate of ATI, regardless of sedation strategy implemented, was nearly 100%. Contrasting this with the successful tracheal intubation rates in patients who are under general anaesthesia of between 94 and 98% [62], this testifies to the efficacy of ATI.

Furthermore, all sedation regimens that were compared with placebo were found to be superior in terms of time to successful ATI. This might be interpreted as sedation facilitating tracheal intubation, regardless of the strategy used. Time to successful tracheal intubation is an important patient‐centred outcome, because the process of ATI may be associated with discomfort, pain and anxiety; thus, reducing the time needed has patient benefits. This contrasts with time to successful tracheal intubation under general anaesthesia, which does not have these drawbacks. Therefore, our findings suggest that patient experience is likely to be better with any sedative than without sedation. Overall, our results suggest that successful ATI is not dependent on the sedatives used, if they are carefully delivered, monitored and titrated. Sedation can have benefits to patient‐centred outcomes.

However, the findings of this systematic review warrant further consideration of dexmedetomidine, either alone or in combination with other drugs, in terms of time to tracheal intubation (although the apparent advantage must be tempered by the need for a 10‐min loading infusion). As a selective ɑ2‐agonist, dexmedetomidine provides effective sedative, anxiolytic and sympatholytic effects, making it a useful drug for procedural sedation in a wide range of settings [63, 64, 65]. While dexmedetomidine is thought to have minimal impact on respiratory drive or pharyngeal collapsibility, recent evidence suggests that this effect may be less marked than previously thought, and could even be similar to propofol [66]. Reducing upper airway patency may impair flexible bronchoscopic visualisation and, in particular, the insertion of a tracheal tube. This could make tracheal intubation more challenging, thus increasing the time to successful tracheal intubation.

In contrast, ketamine maintains upper airway tone and respiratory drive [67]. Therefore, combining the sedative and anxiolytic benefits of dexmedetomidine with the analgesic and ventilatory benefits of ketamine could explain the improved time to tracheal intubation with this combination compared with other techniques. This might also hold true when ketamine was combined with propofol, showing superior time to successful ATI than several other strategies. The low quality of evidence precludes definitive conclusions, but the potential benefits of these combinations are worth consideration. Of note, when combining sedative drugs, it is ideal that these are delivered, monitored and titrated by an independent practitioner and the complications of oversedation are avoided [1]. As noted above, procedural sedation with dexmedetomidine requires a loading infusion of 0.5–1.0 μg kg^‐1^ over 10 min, and therefore this additional time should be factored into the entire ATI procedure [68].

Remifentanil has been seen as an effective and safe sedative for ATI due to its potent analgesia, antitussive effects and rapid onset and offset. Indeed, Difficult Airway Society guidelines suggest that this drug, along with dexmedetomidine, is a suitable choice [1]. However, contrary to previous findings [3], our evidence finds no clear superiority of remifentanil over other drugs. This previous synthesis of evidence neither quantitatively pooled the data, nor compared drugs or strategies, either directly or indirectly, and only included studies up to 2012 [3]. This discrepancy could also be due to heterogeneity in our evidence undermining confidence in any individual drug. However, assuming that our evidence is robust, this suggests that any of the studied drugs could potentially be used, as long as they are given cautiously and safely.

As such, we cannot conclude as yet that there is an ideal sedative for ATI. While dexmedetomidine appears most favourable for some outcomes, novel drugs or strategies may still have a role. In particular, the benzodiazepine remimazolam may have substantial benefits in this setting [69]. It has a relatively rapid onset and offset, and can be reversed with flumazenil in the event of overdose [70]. Further, remimazolam has a favourable haemodynamic profile, therefore, the risk of adverse cardiovascular events may be reduced [70]. Finally, airway tone is thought to be maintained, thereby potentially reducing the risk of airway obstruction and hypoxia, although this is not based on robust data. Evidence in this field is emerging [71, 72], but there are currently insufficient data to draw conclusions.

Our review has limitations. We synthesised a highly heterogeneous group of interventions. We would have liked to stratify our findings based on dosing regimens and routes of administration, but this would have led to results that were too fragile to interpret. There were insufficient data for each group to be able to compare each in a meaningful manner. Furthermore, a wide variety of drugs was used, some of which may have had limited biological plausibility for efficacy, such as magnesium or safety, such as haloperidol. This is a weakness of the network meta‐analysis approach and should alert readers to be aware of potentially spurious results. Pairwise meta‐analyses between interventions, rather than network meta‐analysis could have been used. However, the multitude of possible drug combinations means that the process of selecting each intervention may introduce bias and could exclude drugs that have potential benefits. It may be that narrative synthesis of all evidence is appropriate, but robust quantitative analyses provide a more objective assessment of the literature. The definitions of outcomes included were variable (e.g. time‐to‐performance had variable start and stop points and oxygen desaturation had different thresholds), and although we attempted to correct for this, it may affect our findings. Awake tracheal intubation may be performed using a flexible bronchoscope or a videolaryngoscope, and we have assumed that the results would be similar regardless of the device used. Awake tracheal intubations were performed most commonly by consultants or senior clinicians, and sedation was independently administered in most studies, but these two methodological elements were unclear in a significant proportion of reports. We only assessed a single element of the performance of ATI, and did not account for different topical anaesthetic, oxygenation or procedural strategies that may influence outcomes. Finally, the majority of patients included were in the elective setting, and caution must be exercised when translating these data to the emergency setting.

In conclusion, this systematic review and network meta‐analysis found low‐quality evidence that dexmedetomidine, either alone or in combination with ketamine, might be associated with a reduced time to ATI. Propofol combined with ketamine was also favourable, but there was no other robust evidence for any other differences in outcomes between any sedation strategies used. All sedative regimens were superior to placebo for this outcome. To maximise successful and safe ATI, optimising oxygenation, topical anaesthesia and procedural performance may have more impact than sedation strategy. As such, clinicians should consider using a sedation approach with which they are sufficiently familiar and experienced to use effectively and safely.

## Supporting information


**Appendix S1.** Search strategy.

**Appendix S2.** Network league tables excluding trials at high risk of bias for main outcomes.

**Appendix S3.** GRADE quality of evidence.

**Appendix S4.** Network league tables including all trials for main and other outcomes.

**Figure S1.** Risk of bias assessment of included trials using the revised Cochrane tool.
**Table S1.** Characteristics of included trials.
**Table S2.** Network league tables.
**Table S3.** Full trial details and conclusions from the network meta‐analysis.
**Table S4.** Results from trials not included in the network meta‐analysis.
