# Supplementary material for: Sedation for awake tracheal intubation: A systematic review and network meta‐analysis
Source: Anaesthesia. 2024 Oct 28;80(1):74–84. doi: 10.1111/anae.16452 (PMC11617133; doi:10.1111/anae.16452)
Supplement: Supplementary file 1 — Appendix S1. Search strategy. [file ANAE-80-74-s001.docx]

**Appendix S1.** Search strategies

**EMBASE (967) + (18) In Sept 23**

awake tracheal intubation.mp. or awake tracheal intubation/ OR awake intubation.mp. OR awake fib*.mp. OR awake intub*.mp. OR exp awake tracheal intubation/

AND

endotracheal intubation guide/ or intubation laryngoscope/ or nasotracheal intubation/ or intubation.mp. or fiberoptic tracheal intubation/ or difficult intubation/ or endobronchial intubation/ or respiratory tract intubation/ or intubation/ or endotracheal intubation/ or endotracheal tube/ or reinforced endotracheal tube/ or exp endotracheal intubation/ or endotracheal tube cuff/ or endotracheal tube stylet/ or endotracheal anesthesia/ or endotracheal.mp. or exp endotracheal intubation guide/ or laser-resistant endotracheal tube/ or exp endotracheal intubation/ or bronchoscope tubing/ or bronchoscope/ or bronchoscope.mp. or flexible bronchoscope/ or rigid bronchoscope/ or exp laryngoscopy/ or laryngoscop*.mp. or endotracheal intubation/ or bronchoscopy/ or bronchoscop*.mp. or fiberoptic.mp. or fiber optics/ or laryngoscope/ or videolaryngoscope/ or laryngoscopy/ or videolaryngoscopy/ or videolaryngoscop*.mp. or endotracheal tube/ or endotracheal intubation/ or nasotracheal tube/ or nasotracheal intubation/ or nasotracheal.mp.

AND

sedation.mp. or exp sedation/ or Richmond Agitation Sedation Scale/ or exp deep sedation/ or Ramsay Sedation Scale/ or exp conscious sedation/ or Sedation Agitation Scale/ OR sedation/ or sedative agent/ or sedativ*.mp. or analgesic agent/ or hypnotic.mp. or hypnotic agent/ or anesthesia/ or anaesth*.mp. or fentanyl/ or general anesthesia/ or anesthetic agent/ or general anesth*.mp. or opioid.mp. or opiate/ or anesthesia/ or methohexital/ or midazolam/ or thiopental/ or propofol.mp. or phenol derivative/ or propofol/ or intravenous drug administration/ or diazepam/ or benzodiazepine derivative/ or benzod*.mp. or midazolam/ or benzodiazepine/ or opiate/ or exp midazolam maleate/ or exp midazolam/ or exp lorazepam/ or remimazolam.mp. or exp remimazolam/ or remifentanil.mp. or exp remifentanil/ or exp sufentanil citrate/ or sufentanil.mp. or exp sufentanil/ or sufentanil sublingual tablet system/ or dexmedetomidine.mp. or exp dexmedetomidine/ or exp clonidine derivative/ or exp clonidine/ or clonidine.mp. or exp clonidine/ or exp dexmedetomidine/ or alpha 2 adrenergic receptor stimulating agent/ or ketamine.mp. or exp ketamine/

**Ovid MEDLINE(R) (2048) + (14) In Sept 23**

awake intubation.mp. or awake tracheal.mp. or awake*.mp. or awake tracheal intubation.mp. or awake fiber optic.mp. or awake fiber optic intubation.mp.

AND

intubation.mp. or exp Intubation/ or exp Intubation, Intratracheal/ or exp Respiration, Artificial/ or exp Laryngoscopy/ or exp Laryngoscopes/ or exp Laryngoscopy/ or difficult airway.mp. or Airway Management/or endotracheal.mp. or exp Intubation, Intratracheal/ or exp Anesthesia, Endotracheal/ or exp Airway Management/ or tracheal tube.mp. or exp Bronchoscopy/ or bronchoscop*.mp. or bronchoscopic.mp. or laryngoscopy.mp. or exp Laryngoscopy/ or Laryngeal Diseases/ or laryngoscopic.mp. or exp Fiber Optic Technology/ or nasal intubation.mp. or nasotracheal.mp. or videolaryngoscopy.mp.

AND

exp Deep Sedation/ or exp Conscious Sedation/ or sedation.mp. or sedative.mp. or exp "Hypnotics and Sedatives" or hynoptic.mp. or exp "Anesthesia and Analgesia"/ or exp Analgesia/ or analgesia.mp. or exp Anesthesia/ or anesthesia.mp. or exp Anesthesia, Intravenous/ or exp Anesthesia, General/ or exp Anesthesia, Inhalation/ or exp "Anesthesia and Analgesia"/ or exp Anesthetics, Intravenous/ or anesthe*.mp. or exp Anesthetics/ or exp Anesthesia/ or exp Anesthesia, General/ or fentanyl.mp. or exp Fentanyl/ or sufentanil.mp. or exp Sufentanil or opioid.mp. or Analgesics, Opioid/ or opioid.mp. or exp Analgesics, Opioid/ or opiate.mp. or exp Opiate Alkaloids/ or remifentanil.mp. or exp Remifentanil/ or benzodiazepine.mp. or exp Benzodiazepines/ or midazolam.mp. or exp Midazolam/ or lorazepam.mp. or exp Lorazepam/ or exp Anesthesia, Intravenous/ or propofol.mp. or exp Propofol/ or exp Phenols/ or Anesthetics/ or exp Anesthetics, Intravenous/ or exp "Hypnotics and Sedatives"/ or exp Benzodiazepines/ or remimazolam.mp. or exp Anesthetics, Intravenous or ketamine.mp. or exp Ketamine/ or clonidine.mp. or exp Clonidine or exp Diazepam/ or diazepam.mp. or methohexital.mp. or exp Methohexital/ or Analgesics, Non-Narcotic/ or exp Dexmedetomidine/ or exp Adrenergic alpha-Agonists/ or dexmed*.mp. or exp Adrenergic alpha-2 Receptor Agonists/

**PUBMED (1758) + (31) In Sept 23**

(((((awake tracheal intubation) OR (awake intubat*)) OR (awake fiber optic)) OR (awake fiber optic intubation))

AND

((((((((((((((endotracheal intubation[MeSH Terms]) OR (endotracheal intubations[MeSH Terms])) OR (intratracheal intubation[MeSH Terms])) OR (bronchoscope[MeSH Terms])) OR (bronchoscopes[MeSH Terms])) OR (cable, fiber optic[MeSH Terms])) OR (cables, fiber optic[MeSH Terms])) OR (fiber optic cable[MeSH Terms])) OR (fiber optic cables[MeSH Terms])) OR (fiber optic technologies[MeSH Terms])) OR (laryngoscope[MeSH Terms])) OR (laryngoscopes[MeSH Terms])) OR (airway management[MeSH Terms])) OR ((((((((((intubat*) OR (nasal intubation)) OR (nasal tracheal intubation)) OR (difficult airway)) OR (difficult intubation)) OR (video laryngoscopy)) OR (video laryngoscope)) OR (fiber optic)) OR (fiberoptic*)) OR (bronchoscop*))))

AND

((((((((((((((((((((((((((((((((((((conscious sedation[MeSH Terms]) OR (deep sedation[MeSH Terms])) OR (deep sedations[MeSH Terms])) OR (moderate sedation[MeSH Terms])) OR (effect, sedative[MeSH Terms])) OR (analgesia and anesthesia[MeSH Terms])) OR (agents, anesthetic[MeSH Terms])) OR (adjuvants, anesthetic[MeSH Terms])) OR (adjuvants, anesthesia[MeSH Terms])) OR (anesthesia[MeSH Terms])) OR (benzodiazepine compounds[MeSH Terms])) OR (benzodiazepine[MeSH Terms])) OR (diprivan[MeSH Terms])) OR (anesthesia, intravenous[MeSH Terms])) OR (anesthesias, intravenous[MeSH Terms])) OR (anesthetics, intravenous[MeSH Terms])) OR (abbott brand of propofol[MeSH Terms])) OR (alpha brand of propofol[MeSH Terms])) OR (astra brand of propofol[MeSH Terms])) OR (astrazeneca brand of propofol[MeSH Terms])) OR (braun brand of propofol[MeSH Terms])) OR (propofol)) OR (benzodiazepine)) OR (midazolam)) OR (lorazepam)) OR (remifentanil)) OR (analgesics, opioid[MeSH Terms])) OR (dexmed*)) OR (anaesthe*)) OR (anesthe*)))) OR (((((((hypnotics) OR (sufentanil)) OR (remimazolam)) OR (clonidine)) OR (ketamine)) OR (methohexital)) OR (thiopentone)))) OR ((((((((((((((((((hypnotics and sedatives[MeSH Terms]) OR (effects, sedative[MeSH Terms])) OR (sedative effect[MeSH Terms])) OR (sedative effects[MeSH Terms])) OR (fentanyl[MeSH Terms])) OR (alkaloids, opiate[MeSH Terms])) OR (anesthetic hypnosis[MeSH Terms])) OR (anesthetic hypnoses[MeSH Terms])) OR (hydrochloride, midazolam[MeSH Terms])) OR (maleate, midazolam[MeSH Terms])) OR (midazolam[MeSH Terms])) OR (midazolam hydrochloride[MeSH Terms])) OR (midazolam maleate[MeSH Terms])) OR (ahp brand of lorazepam[MeSH Terms])) OR (apo lorazepam[MeSH Terms])) OR (apotex brand of lorazepam[MeSH Terms])) OR (baxter brand of lorazepam[MeSH Terms])) OR (ct arzneimittel brand of lorazepam[MeSH Terms]))) OR (((clonidine[MeSH Terms]) OR (dexmedetomidine[MeSH Terms])) OR (citrate, sufentanil[MeSH Terms])))

**CENTRAL (96) + (0) SEPT 23**

ID Search

#1 awake

#2 awake fiber optic

#3 awake tracheal

#4 Awake intubation

#5 MeSH descriptor: [Intubation, Intratracheal] explode all trees

#6 laryngoscopy

#7 laryngoscope

#8 videolaryngoscope

#9 videolaryngoscopy

#10 fiber optic

#11 fiberoptic

#12 difficult airway

#13 MeSH descriptor: [Conscious Sedation] explode all trees

#14 MeSH descriptor: [Airway Management] explode all trees

#15 MeSH descriptor: [Analgesics, Opioid] explode all trees

#16 MeSH descriptor: [Benzodiazepines] explode all trees

#17 MeSH descriptor: [Propofol] explode all trees

#18 MeSH descriptor: [Clonidine] explode all trees

#19 MeSH descriptor: [Dexmedetomidine] explode all trees

#20 MeSH descriptor: [Fentanyl] explode all trees

#21 MeSH descriptor: [Remifentanil] explode all trees

#22 MeSH descriptor: [Ketamine] explode all trees

#23 MeSH descriptor: [Midazolam] explode all trees

#24 MeSH descriptor: [Diazepam] explode all trees

#25 MeSH descriptor: [Lorazepam] explode all trees

#26 MeSH descriptor: [Laryngoscopy] explode all trees

#27 MeSH descriptor: [Optical Fibers] explode all trees

#28 MeSH descriptor: [Intubation] explode all trees

#29 #1 OR #2 OR #3 OR #4

#30 #5 OR #6 OR #7 OR #8 OR #9 OR #10 OR #11 OR #12 OR #14 OR #26 OR #27 OR #28

#31 #13 OR #15 OR #16 OR #17 OR #18 OR #19 OR #20 OR #21 OR #22 OR #23 OR #24 OR #25

#32 #29 AND #30 AND #31
