# Supplementary material for: Sedation for awake tracheal intubation: A systematic review and network meta‐analysis
Source: Anaesthesia. 2024 Oct 28;80(1):74–84. doi: 10.1111/anae.16452 (PMC11617133; doi:10.1111/anae.16452)
Supplement: Supplementary file 3 — Appendix S3. GRADE quality of evidence. [file ANAE-80-74-s005.docx]

**Appendix S3** GRADE quality of evidence.

| **Outcome** | **Limitations** | **Indirectness** | **Imprecision** | **Inconsistency** | **Publication bias** | **Total number of participants** | **Conclusion** | **Quality of evidence** |
| --- | --- | --- | --- | --- | --- | --- | --- | --- |
| *Overall awake tracheal intubation success rate (%)* | Serious limitations^a^ | No serious indirectness | Serious imprecision^b^ | No serious inconsistency | No serious publication bias | 2246 | No differences between interventions | Low quality (⊕⊕) |
| *Time to tracheal intubation (s)* | Serious limitations^a^ | No serious indirectness | Serious imprecision^b^ | No serious inconsistency | No serious publication bias | 1745 | Placebo inferior to all other interventions  Dexmedetomidine superior to fentanyl  Dexmedetomidine + ketamine superior to alfentanil, dexmedetomidine, dexmedetomidine + propofol, fentanyl, fentanyl + ketamine, fentanyl + propofol, midazolam, midazolam + clonidine, midazolam + dexmedetomidine, midazolam + fentanyl, midazolam + propofol, midazolam + remifentanil, midazolam + sufentanil, placebo, propofol, remifentanil and sufentanil  Ketamine + propofol superior to alfentanil, dexmedetomidine, dexmedetomidine + propofol, fentanyl, fentanyl + ketamine, fentanyl + propofol, midazolam, midazolam + clonidine, midazolam + dexmedetomidine, midazolam + fentanyl, midazolam + propofol, midazolam + remifentanil, midazolam + sufentanil, placebo, propofol, remifentanil and sufentanil  Magnesium sulphate superior to alfentanil, dexmedetomidine, dexmedetomidine + propofol, fentanyl, fentanyl + ketamine, fentanyl + propofol, midazolam, midazolam + clonidine, midazolam + dexmedetomidine, midazolam + fentanyl, midazolam + propofol, placebo and propofol  Midazolam + dexmedetomidine superior to midazolam  Remifentanil superior to dexmedetomidine, fentanyl, midazolam + fentanyl and placebo | Low quality (⊕⊕) |
| *Incidence of arterial oxygen desaturation (%)* | Serious limitations^a^ | No serious indirectness | Serious imprecision^b^ | No serious inconsistency | No serious publication bias | 1813 | Dexmedetomidine superior to fentanyl and propofol  Magnesium sulphate superior to fentanyl, midazolam, midazolam + fentanyl, midazolam + propofol, placebo, propofol and remifentanil | Low quality (⊕⊕) |
| *Need for rescue analgesia (%)* | No limitations | No serious indirectness | Serious imprecision^b^ | No serious inconsistency | Serious publication bias^d^ | 578 | Dexmedetomidine and remifentanil superior to placebo | Low quality (⊕⊕) |
| *Time to conduct fibrescopy (s)* | Serious limitations^a^ | No serious indirectness | Serious imprecision^b^ | No serious inconsistency | No serious publication bias | 351 | Data not available for placebo  No differences between interventions | Low quality (⊕⊕) |
|  |  |  |  |  |  |  |  |  |
|  |  |  |  |  |  |  |  |  |
|  |  |  |  |  |  |  |  |  |
| *Incidence of adverse cardiovascular events (%)* | Serious limitations^a^ | No serious indirectness | Serious imprecision^b^ | No serious inconsistency | No serious publication bias | 1323 | Alfentanil superior to dexmedetomidine, dexmedetomidine + fentanyl, fentanyl + propofol, midazolam, midazolam + fentanyl, placebo and sufentanil  Alfentanil, dexmedetomidine, dexmedetomidine + propofol, fentanyl, fentanyl + propofol, midazolam, midazolam + dexmedetomidine, midazolam + fentanyl, midazolam + sufentanil, placebo, propofol, remifentanil, remifentanil + propofol and sufentanil superior to dexmedetomidine + fentanyl  Propofol superior to fentanyl + propofol and midazolam | Low quality (⊕⊕) |

^a^In many comparisons between different interventions, some concerns or major concerns were present for the overall risk of bias. Final decision to rate down quality of evidence for serious limitations.

^b^Evidence of serious imprecision as some or most comparisons between different interventions have a confidence interval that extends into clinically important or unimportant effects. Final decision to rate down quality of evidence for serious imprecision.

^c^Evidence of serious inconsistency as most comparisons between different interventions have variability of direct and indirect effects in relation to a clinically important size of effect. Final decision to rate down quality of evidence for serious inconsistency.

^d^Final decision to rate down quality of evidence for serious publication bias.
