# Supplementary material for: Sedation for awake tracheal intubation: A systematic review and network meta‐analysis
Source: Anaesthesia. 2024 Oct 28;80(1):74–84. doi: 10.1111/anae.16452 (PMC11617133; doi:10.1111/anae.16452)
Supplement: Supplementary file 5 — Figure S1. Risk of bias assessment of included trials using the revised Cochrane tool. Table S1. Characteristics of included trials. Table S2. Network league tables. Table S3. Full trial details and conclusions from the network meta‐analysis. Table S4. Results from trials not included in the network meta‐analysis. [file ANAE-80-74-s003.docx]

**Table S1** Characteristics of the included trials.

| **Reference** | **Group (n)** | **Indication for awake tracheal intubation** | **Method of sedation administration** | **Same or separate sedationist and airway operator(s)** | **Experience of airway operator** | **Topicalisation** |
| --- | --- | --- | --- | --- | --- | --- |
| Abdel Hamid et al 2008 [14] | Remifentanil (15)  Remifentanil + propofol (15) | Patients scheduled for elective surgery under general anaesthesia with anticipated difficult airway  ASA 1–2 Mallampati score not specified  Age group 18 years or older | Remifentanil: 0.05 μg.kg^-1^ over 10 min followed by infusion at a rate of 0.05 μg.kg.min^-1^  Remifentanil + propofol: remifentanil 0.05 μg.kg^-1^ over 10 min followed by infusion at a rate of 0.05 μg.kg.min^-1^ and infusion of propofol at a rate of 50 μg.kg.min^-1^ | Separate | Experienced consultant anaesthetist | Lidocaine |
| Acharya et al 2022 [55] | Dexmedetomidine (34) Dexmedetomidine + fentanyl (34) | Patients scheduled for elective dental or oral cancer surgery under general anaesthesia ASA 1–2 Mallampati score not specified Age group 18–65 years old | Dexmedetomidine: 1 μg.kg^-1^ over 10 min Dexmedetomidine + fentanyl: dexmedetomidine 1 μg.kg^-1^ and fentanyl 1 µg.kg^-1^ over 10 min | Not specified | Not specified | Lidocaine  Oxymetazoline |
| Agrawal et al 2014 [15] | Dexmedetomidine (30)  Midazolam + fentanyl (30) | Patients scheduled for elective surgery under general anaesthesia with anticipated difficult airway  ASA 1–2  Mallampati score not specified  Age group 18 years or older | Dexmedetomidine: 1 μg.kg^-1^ over 10 min  Midazolam + fentanyl: midazolam 1 mg bolus and infusion of fentanyl at a rate of 1 μg.kg^-1^ | Separate | Not specified | Lidocaine  Xylometazoline |
| Baiju et al 2020 [26] | Fentanyl (20)  Dexmedetomidine (20) | Patients scheduled for elective surgery under general anaesthesia with anticipated difficult airway  ASA 1–3  Mallampati score not specified  Age group 20–65 years old | Fentanyl: 2 μg.kg^-1^ over 10 min  Dexmedetomidine: 1 μg.kg^-1^ over 10 min | Separate | Expert anaesthetist | Lidocaine |
| Bano et al 2019 [37] | Midazolam (20)  Midazolam + dexmedetomidine (20)  Midazolam + clonidine (20) | Patients scheduled for elective surgery under general anaesthesia with anticipated difficult airway  ASA 1–2  Mallampati score not specified  Age group 18–60 years old | Midazolam: 0.04 mg.kg^-1^ bolus followed by 0.05 mg.kg^-1^ over 10 min and then infusion at a rate of 0.05 mg.kg.^-1^.h^-1^  Midazolam + dexmedetomidine: midazolam 0.04 mg.kg^-1^ bolus followed by dexmedetomidine 1 μg.kg^-1^ over 10 min and then infusion at a rate of 0.5 µg.kg^-1^.h^-1^  Midazolam + clonidine: midazolam 0.04 mg.kg^-1^ bolus followed by clonidine 2 μg.kg^-1^ over 10 min and then infusion at a rate of 2 µg.kg^-1^.h^-1^ | Separate | Consultant anaesthetist | Lidocaine  Xylometazoline |
| Bergese et al 2010 [48] | Placebo (50)  Dexmedetomidine (55) | Patients scheduled for elective surgery under general anaesthesia with anticipated difficult airway  ASA 1–4  Mallampati score 1–4  Age group 18 years or older | Dexmedetomidine: 1 mcg.kg^-1^ over 10 min followed by infusion at a rate of 0.7mcg.kg^-1^.hr^-1^ | Not specified | Not specified | Lidocaine |
| Bergese et al 2010 2 [57] | Midazolam (24)  Midazolam + dexmedetomidine (31) | Patients scheduled for elective surgery under general anaesthesia with anticipated difficult airway  ASA PS not specified  Mallampati score 1–4  Age group 18 years or older | Midazolam: 0.05 mg.kg^-1^ bolus followed by 0.05 mg.kg^-1^ as needed  Midazolam + dexmedetomidine: midazolam 0.02 mg.kg^-1^ bolus followed by dexmedetomidine 1 μg.kg^-1^ over 15 min and then infusion titrated to a rate of 0.7 μg.kg^-1^.hr^-1^ | Not specified | Not specified | Lidocaine |
| Cattano et al 2012 [58] | Midazolam + remifentanil (17)  Midazolam + dexmedetomidine (13) | Patients scheduled for elective surgery under general anaesthesia with anticipated difficult airway  ASA 1–3  Mallampati score 1–4 Age group 18 years or older | Midazolam + remifentanil: midazolam 2 mg bolus followed by remifentanil 0.75 mcg.kg^-1^ over 10 min and infusion at a rate of 0.075 μg.kg^-1^.min^-1^  Midazolam + dexmedetomidine: midazolam 2 mg bolus followed by dexmedetomidine 0.4 μg.kg^-1^ over 10 min and then infusion at a rate of 0.7 μg.kg^-1^.hr^-1^ | Separate | Resident trainees | Lidocaine  Oxymetazoline |
| Chalam 2015 [59] | Diazepam + propofol (50)  Diazepam + dexmedetomidine (50) | Patients scheduled for elective cervical spine surgery under general anaesthesia  ASA 1–2  Mallampati score not specified  Age group 20–60 years old | Diazepam + propofol: oral diazepam 10 mg bolus followed by propofol 1 mg.kg^-1^ over 5 min  Diazepam + dexmedetomidine: oral diazepam 10 mg bolus followed by dexmedetomidine 1 μg.kg^-1^ over 10 min and then 0.5 μg.kg^-1^ as needed | Same | Senior anaesthetist | Lidocaine |
| Chaudhary et al 2021 [60] | Fentanyl (31)  Nalbuphine (31) | Patients scheduled for elective surgery under general anaesthesia with anticipated difficult airway  ASA 1–2  Mallampati score 1 or 2  Age group 20–60 years old | Fentanyl: 2 µg.kg^-1^ over 10 min  Nalbuphine: 0.2 mg.kg^-1^ over 10 min | Separate | Not specified | Lidocaine  Xylometazoline |
| Chopra et al 2016 [61] | Placebo (50)  Dexmedetomidine (50) | Patients simulating cervical spine injury scheduled for elective surgery under general anaesthesia  ASA 1–2  Mallampati score 1–3  Age group 18–65 years old | Dexmedetomidine: oral alprazolam 0.5 mg bolus followed by dexmedetomidine 1 µg.kg^-1^ over 10 min and then infusion at a rate of 0.7 µg.kg^-1^.h^-1^ | Separate | Experienced anaesthetist | Lidocaine  Xylometazoline |
| Chu et al 2010 [16] | Fentanyl (14)  Dexmedetomidine (16) | Patients scheduled for elective oral cancer surgery under general anaesthesia  ASA 1–3 Mallampati score not specified Age group 18 years or older | Fentanyl: 1 µg.kg^-1^ over 10 min  Dexmedetomidine: 1 µg.kg^-1^ over 10 min | Separate | Experienced anaesthetists | Cocaine  Lidocaine |
| Dey et al 2019 [17] | Propofol (45)  Dexmedetomidine (45) | Patients scheduled for elective surgery under general anaesthesia with anticipated difficult airway  ASA 1–2  Mallampati score 3–4  Age group 18–60 years old | Propofol: 0.1 mg.kg^-1^.min^-1^ bolus followed by infusion at a rate of 0.025 mg.kg^-1^.min^-1^ and then titrated up to 0.075 mg.kg^-1^.min^-1^  Dexmedetomidine: 1 µg.kg^-1^ over 15 min followed by infusion at a rate of 0.2 µg.kg^-1^.h^-1^ and then titrated up to 0.7 µg.kg^-1^.h^-1^ | Not specified | Not specified | Lidocaine  Oxymetazoline |
| Eftekharian et al 2015 [18] | Midazolam + fentanyl + remifentanil (18)  Midazolam + fentanyl + ketamine (18)  Midazolam + fentanyl + propofol (18) | Patients scheduled for elective temporomandibular joint ankylosis surgery under general anaesthesia  ASA 1  Mallampati score 4  Age group 15–60 years old | Midazolam + fentanyl + remifentanil: midazolam 0.05 mg.kg^-1^ bolus and fentanyl 100 µg.kg^-1^ bolus, followed by remifentanil 0.75 µg.kg^-1^ over 30 sec and then infusion a rate of 0.05 µg.kg^-1.^min^-1^  Midazolam + fentanyl + ketamine: midazolam 0.05 mg.kg^-1^ bolus and fentanyl 100 µg.kg^-1^ bolus, followed by ketamine 0.25 mg.kg ^-1^ over 30 sec and then infusion at a rate of 20 mg.h^-1^  Midazolam + fentanyl + propofol: midazolam 0.05mg.kg^-1^ bolus and fentanyl 100 µg.kg^-1^ bolus, followed by propofol 0.5 mg.kg^-1^ over 30 sec and then infusion at a rate of 25 µg.kg^-1.^min^-1^ | Same | Experienced anaesthetists | Lidocaine  Phenylephrine |
| El Mourad et al 2019 [19] | Dexmedetomidine + propofol (40)  Ketamine + propofol (40) | Patients scheduled for elective laryngeal mass biopsy under general anaesthesia  ASA 1–3  Mallampati score not specified  Age group 18–60 years old | Dexmedetomidine + propofol: oral diazepam 10 mg bolus followed by dexmedetomidine 1 µg.kg^-1^ over 10 min and then infusion at a rate of 0.5 µg.kg^-1.^h^-1^, and propofol 1 µg.kg^-1^ over 10 min followed by 0.5 µg.kg^-1.^h^-1^ infusion  Ketamine + propofol: oral diazepam 10 mg bolus followed by ketamine 2 mg.ml^-1^ and propofol 4 mg.ml^-1^ over 10 min and then infusion at a rate of 0.125 ml.kg^-1^.h^-1^ | Separate | Senior anaesthetist | Ephedrine  Lidocaine  Xylometazoline |
| El-Samahy et al 2008 [20] | Midazolam + remifentanil (25)  Midazolam + dexmedetomidine (25) | Patients scheduled for elective oral cancer surgery under general anaesthesia  ASA 1–2  Mallampati score 3–4  Age group 25–60 years old | Midazolam + remifentanil: midazolam 0.02 mg.kg^-1^ and remifentanil 0.75 µg.kg^-1.^ over 30 sec followed by infusion at a rate of 0.075 µg.kg^-1^.min^-1^  Midazolam + dexmedetomidine: midazolam 0.02 mg.kg^-1^ and dexmedetomidine 1 µg.kg^-1^ over 10 min followed by infusion at a rate of 0.7 µg.kg^-1^.h^-1^ | Not specified | Not specified | Lidocaine |
| Elgebaly et al 2014 [21] | Placebo (40)  Midazolam (40)  Magnesium sulphate (40) | Patients scheduled for elective surgery under general anaesthesia with anticipated difficult airway  ASA PS not specified  Mallampati score 1–4  Age group 22–66 years old | Midazolam: 0.07 mg.kg^-1^ over 10 min  Magnesium sulphate: 45 mg.kg^-1^ over 10 min | Separate | Consultant anaesthetist | Lidocaine  Oxymetazoline |
| El Sharkawy 2019 [22] | Dexmedetomidine + ketamine (30)  Ketamine + propofol (30) | Patients scheduled for elective surgery under general anaesthesia with anticipated difficult airway  ASA 1–2  Mallampati score not specified  Age group 18–60 years old | Dexmedetomidine + ketamine: ketamine 0.5 mg.kg^-1^ and dexmedetomidine 1 µg.kg^-1^ over 10 min followed by infusion of ketamine at a rate of 0.5 mg.kg^-1^ and dexmedetomidine at a rate of 1 µg.kg^-1^  Ketamine + propofol: ketamine 0.5 mg.kg^-1^ and propofol 1 mg.kg^-1^ over 10 min followed by infusion of ketamine at a rate of 0.5 mg.kg^-1^ and propofol at a rate of 1 mg.kg^-1^ | Not specified | Not specified | Lidocaine |
| Gupta et al 2012 [23] | Propofol (23)  Dexmedetomidine + propofol (23) | Patients scheduled for elective temporomandibular joint ankylosis surgery under general anaesthesia  ASA 1–2  Mallampati score not specified  Age group 14–38 years old | Propofol: Not specified  Dexmedetomidine + propofol: dexmedetomidine 1 µg.kg^-1^ over 10 min followed by infusion of propofol at an unspecified rate | Separate | Consultant anaesthetist | Lidocaine  Xylometazoline |
| Hu et al 2013 [24] | Dexmedetomidine (20)  Remifentanil (20) | Patients undergoing elective maxillofacial cancer or fracture surgery under general anaesthesia  ASA 1–3  Mallampati score 3–4  Age group 18 years or older | Dexmedetomidine: 1.5 µg.kg^-1^ over 10 min followed by infusion at a rate of 0.7 µg.kg^-1^.hr^-1^  Remifentanil: TCI 2.5–3.5 ng.ml^-^ | Separate | Consultant anaesthetist | Ephedrine  Lidocaine  Nitrofurazone |
| Jafari et al 2020 [25] | Dexmedetomidine (30)  Alfentanil (30) | Patients scheduled for urological surgery under general anaesthesia  ASA 1–2  Mallampati score 1–2  Age group 30–55 years old | Dexmedetomidine: 1 µg.kg^-1^ over 10 min followed by infusion at a rate of 0.5 µg.kg^-1^.hr^-1^  Alfentanil: 20 µg.kg^-1^ over 60–90 sec followed by 10 µg.kg^-1^ repeated every 1–2 min | Separate | Experienced anaesthetists | Lidocaine |
| Kaur et al 2019 [27] | Dexmedetomidine + ketamine (50)  Dexmedetomidine + propofol (50) | Patients scheduled for elective surgery under general anaesthesia with anticipated difficult airway  ASA 1–2 Mallampati score not specified Age group 18–60 years old | Dexmedetomidine + ketamine: dexmedetomidine 1 µg.kg^-1^ over 10 min followed by ketamine 0.25 mg.kg^-1^ bolus  Dexmedetomidine + propofol: dexmedetomidine 1 µg.kg^-1^ over 10 min followed by propofol 1 mg.kg^-1^ bolus | Not specified | Not specified | Lidocaine  Xylometazoline |
| Kumar et al 2017 [28] | Fentanyl + propofol (30)  Midazolam + fentanyl (30) | Patients scheduled for elective surgery under general anaesthesia with anticipated difficult airway  ASA 1–2 Mallampati score not specified  Age group 18–60 years old | Fentanyl + propofol: fentanyl 1 µg.kg^-1^ and propofol 1 mg.kg^-1^ bolus  Midazolam + fentanyl: fentanyl 1 µg.kg^-1^ and midazolam 0.03 mg.kg^-1^ bolus | Not specified | Not specified | Lidocaine  Xylometazoline |
| Lallo et al 2009 [29] | Remifentanil (30)  Propofol (30) | Patients scheduled for elective otolaryngological cancer surgery under general anaesthesia  ASA 1–3  Mallampati score 1–4 Age group 18 years or older | Remifentanil: TCI 0.5 ng.ml^-1^ titrated up to 1.5 ng.ml^-1^  Propofol: TCI 1 µg.ml^-1^ titrated up to 2.5 µg.ml^-1^ | Same | Experienced anaesthetists | Lidocaine  Naphazoline |
| Lee et al 2013 [30] | Remifentanil (25)  Propofol (25) | Patients scheduled for elective surgery under general anaesthesia with anticipated difficult airway ASA 1–2 Mallampati score not specified  Age group 18 years or older | Remifentanil: TCI 0.5 ng.ml^-1^ titrated up to 1 ng.ml^-1^  Propofol: TCI 0.5 µg.ml^-1^ titrated up to 1 µg.ml^-1^ | Not specified | Consultant anaesthetists | Not defined |
| Li et al 2015 [31] | Midazolam + dexmedetomidine (25)  Midazolam + sufentanil (25) | Patients scheduled for elective maxillofacial fracture surgery under general anaesthesia  ASA 1–2  Mallampati score 3–4 Age group 18 years or older | Midazolam + dexmedetomidine: midazolam 0.02 mg.kg^-1^ bolus followed by dexmedetomidine 0.5 µg.kg^-1^ over 10 min and then infusion at a rate of 0.25 µg.kg^-1^.hr^-1^  Midazolam + sufentanil: midazolam 0.02 mg.kg^-1^ bolus followed by sufentanil 0.2 µg.kg^-1^ over 10 min and then infusion at a rate of 0.1 µg.kg^-1^.hr^-1^ | Separate | Experienced consultant anaesthetists | Lidocaine  Tetracaine |
| Liu et al 2015 [32] | Dexmedetomidine (45)  Remifentanil (45) | Patients scheduled for elective surgery under general anaesthesia with anticipated difficult airway  ASA 1–2  Mallampati score not specified  Age group 18 years or older | Dexmedetomidine: 1 µg.kg^-1^ over 10 min followed by infusion at a rate of 0.3 µg.kg^-1^.hr^-1^  Remifentanil: 0.15 µg.kg^-1^.min^-1^ titrated up to 0.75 µg. kg^-1^.min^-1^ over 5 min followed by infusion at a rate of 0.1 µg.kg^-1^.min^-1^ | Separate | Resident trainees | Lidocaine |
| Masoud et al 2013 [33] | Midazolam + fentanyl (25)  Midazolam + propofol (25)  Dexmedetomidine (25) | Patients scheduled for elective surgery under general anaesthesia with anticipated difficult airway  ASA 1–2  Mallampati score not specified  Age group 18 years or older | Midazolam + fentanyl: midazolam 0.05 mg.kg^-1^ bolus followed by infusion of fentanyl at a rate of 0.2 µg.kg^-1^.min^-1^  Midazolam + propofol: midazolam 0.05mg.kg^-1^ bolus followed by infusion of propofol at a rate of 75 µg.kg^-1^ Dexmedetomidine: 1 µg.kg^-1^ over 10 min followed by infusion at a rate of 0.5–0.7 µg.kg^-1^.hr^-1^ | Separate | Expert endoscopist | Lidocaine |
| Meena et al 2021 [34] | Midazolam (30)  Dexmedetomidine (30) | Patients scheduled for elective maxillofacial fracture surgery under general anaesthesia  ASA 2–3 Mallampati score not specified  Age group 18–50 years old | Midazolam: 0.05 mg.kg^-1^ over 10 min followed by infusion at a rate of 0.1 mg.kg^-1^.hr^-1^ titrated up to 0.2 mg.kg^-1^.hr^-1^  Dexmedetomidine: 1 µg.kg^-1^ over 10 min followed by infusion at a rate of 0.2 µg.kg^-1^.hr^-1^ titrated up to 0.7 µg.kg^-1^.hr^-1^ | Not specified | Not specified | Lidocaine |
| Mirkheshti et al 2017 [35] | Propofol (32)  Propofol + dexmedetomidine (31)  Propofol + perineural dexmedetomidine (32) | Patients scheduled for elective surgery under general anaesthesia with anticipated difficult airway  ASA 1–2  Mallampati score not specified  Age group 18–65 years old | Propofol: 30 mg.kg^-1^ bolus  Propofol + dexmedetomidine: propofol 30 mg.kg^-1^ and dexmedetomidine 1 µg.kg^-1^ bolus  Propofol + perineural dexmedetomidine: propofol 30 mg.kg^-1^ and dexmedetomidine 1 µg.kg-^1^ bolus | Separate | Experienced anaesthetists | Lidocaine |
| Mondal et al 2015 [36] | Alprazolam + fentanyl (30)  Alprazolam + dexmedetomidine (30) | Patients scheduled for elective abdominal surgery under general anaesthesia  ASA 1–2  Mallampati score 1–2  Age group 20–60 years old | Alprazolam + fentanyl: oral alprazolam 0.5 mg followed by fentanyl 2 µg.kg^-1^ over 10 min  Alprazolam + dexmedetomidine: oral alprazolam 0.5 mg followed by dexmedetomidine 1 µg.kg^-1^ over 10 min | Separate | Consultant anaesthetists | Lidocaine  Xylometazoline |
| Niyogi et al 2017 [38] | Placebo (28)  Dexmedetomidine (28) | Patients undergoing elective cervical fixation surgery for cervical spondylotic myelopathy under general anaesthesia  ASA 1–2 Mallampati score not specified  Age group 18–50 years old | Dexmedetomidine: 1 µg.kg^-1^ over 10 min followed by infusion at a rate of 0.5 µg.kg^-1^.h^-1^ | Not specified | Experienced anaesthetist | Lidocaine  Xylometazoline |
| Puchner et al 2002 [39] | Midazolam + fentanyl (37)  Midazolam + remifentanil (37) | Patients scheduled for elective oral and maxillofacial surgery under general anaesthesia  ASA 1–2 Mallampati score not specified Age group 19–75 years old | Midazolam + fentanyl: oral midazolam 7.5–15 mg followed by fentanyl 0.5 µg.kg^-1^ and then midazolam 1 mg or 10 mg  Midazolam + remifentanil: oral midazolam 7.5–15 mg followed by infusion of remifentanil at a rate of 0.1–0.5 mg.kg^-1^.min^-1^ | Not specified | Not specified | Lidocaine  Xylometazoline |
| Rai et al 2008 [40] | Midazolam + propofol (10)  Midazolam + remifentanil (14) | Patients scheduled for elective surgery under general anaesthesia with anticipated difficult airway  ASA 1–3 Mallampati score not specified Age group 18 years or older | Midazolam + propofol: midazolam 1–2 mg bolus followed by target concentration of propofol 1 µg.ml^-1^  Midazolam + remifentanil: Midazolam 1-2 mg followed by remifentanil TCI 3 ng.ml^-1^ | Separate | Experienced anaesthetists | Cocaine  Lidocaine |
| Rajan et al 2018 [41] | Fentanyl (20)  Dexmedetomidine (20) | Patients scheduled for head and neck surgery under general anaesthesia  ASA 1–3 Mallampati score not specified  Age group 18 years or older | Fentanyl: 2 µg.kg^-1^ bolus  Dexmedetomidine: 1 µg.kg^-1^ bolus | Not specified | Not specified | Lidocaine |
| Randell et al 1990 [42] | Diazepam (18)  Diazepam + alfentanil (16) | Patients scheduled for elective surgery under general anaesthesia with anticipated difficult airway  ASA 1–2 Mallampati not specified Age group 18 years or older | Diazepam: 0.1 mg.kg^−1^ bolus  Diazepam + alfentanil: diazepam 0.1 mg.kg^−1^ bolus followed by alfentanil 20 µg.kg^-1^ bolus and diazepam 2.5 mg if needed | Not specified | Not specified | Lidocaine |
| Sachan et al 2022 [56] | Midazolam + fentanyl (30)  Dexmedetomidine (30) | Patients scheduled for elective oral and maxillofacial surgery under general anaesthesia ASA 1–2 Mallampati not specified  Age group 18–55 years old | Midazolam + fentanyl: midazolam 0.05 mg.kg^-1^ bolus followed by fentanyl 1 µg.kg^-1^ bolus and midazolam 0.02 mg.kg^−1^ if needed Dexmedetomidine: 1 µg.kg^-1^ over 10 min followed by infusion at a rate of 0.5–0.7 µg.kg^-1^.hr^-1^ | Separate | Not specified | Lidocaine |
| Sayeed et al 2013 [43] | Midazolam + fentanyl (16)  Dexmedetomidine (16) | Patients scheduled for elective surgery under general anaesthesia with anticipated difficult airway ASA 1–3  Mallampati score not specified Age group 18 years or older | Midazolam + fentanyl: midazolam 1 mg bolus followed by fentanyl 1 µg.kg^-1^ bolus  Dexmedetomidine: 1 µg.kg^-1^ over 10 min | Separate | Consultant anaesthetists | Lidocaine  Oxymetazoline |
| Shah et al 2013 [44] | Haloperidol + midazolam (22)  Haloperidol + dexmedetomidine (25) | Patients scheduled for cardiac surgery under general anaesthesia with anticipated nondifficult airway  ASA 2–3  Mallampati score not specified Age group 18 years or older | Haloperidol + midazolam: oral haloperidol 5 mg followed by midazolam 0.05 mg.kg^-1^ bolus  Haloperidol + dexmedetomidine: oral haloperidol 5 mg followed by dexmedetomidine 1 µg.kg^-1^ over 5 min and then infusion at a rate of 0.1 µg.kg^-1^.hr^-1^ | Separate | Experienced anaesthetists | Lidocaine  Xylometazoline |
| Shen et al 2014 [45] | Dexmedetomidine (20)  Sufentanil (20) | Patients scheduled for elective surgery with ankylosis of cervical vertebra fracture, facial scar, temporomandibular joint ankylosis and tongue neoplasm under general anaesthesia  ASA 1–3  Mallampati score 3–4  Age group 19–65 years old | Dexmedetomidine: 1 µg.kg^-1^ over 10 min followed by infusion at a rate of 0.5 µg.kg^-1^.hr^-1^  Sufentanil: TCI 0.3 ng/ml^-1^ | Not specified | Not specified | Lidocaine |
| Sinha et al 2014 [46] | Dexmedetomidine (30)  Dexmedetomidine + ketamine (30) | Patients scheduled for elective surgery under general anaesthesia  ASA 1–2 Mallampati score not specified  Age group 18-60 years old | Dexmedetomidine: 1 µg.kg^-1^ over 10 min followed by infusion at a rate of 0.5 µg.kg^-1^.hr^-1^  Dexmedetomidine + ketamine: dexmedetomidine 1 µg.kg^-1^ over 10 min followed by infusion at a rate of 0.5 µg.kg^-1^.hr^-1^, and ketamine 15 mg bolus followed by infusion at a rate of 20 mg.hr^-1^ | Separate | Experienced anaesthetists | Lidocaine  Xylometazoline |
| Soliman et al 2013 [47] | Fentanyl + propofol (20)  Dexmedetomidine (20) | Patients scheduled for elective surgery under general anaesthesia with anticipated difficult airway ASA 1–3 Mallampati score 3–4 Age group 19–59 years old | Fentanyl + propofol: infusion of fentanyl at a rate of 0.5 µg.kg^-1^.hr^-1^ and propofol at a rate of 30 µg.kg^-1^.min^-1^  Dexmedetomidine: 1 µg.kg^-1^ over 10 min followed infusion at a rate of 0.7 µg.kg^-1^.hr^-1^ | Separate | Experienced anaesthetists | Lidocaine |
| Tsai et al 2010 [49] | Propofol (20)  Dexmedetomidine (20) | Patients scheduled for elective oral cancer surgery under general anaesthesia  ASA 1–3 Mallampati not specified  Age group 18 years or older | Propofol: TCI 3 µg.ml^-1^  Dexmedetomidine: 1 µg.kg^-1^ over 10 min | Separate | Experienced anaesthetists | Cocaine  Lidocaine |
| Verma et al 2021 [50] | Fentanyl + ketamine (30)  Dexmedetomidine (30) | Patients scheduled for elective surgery under general anaesthesia with anticipated difficult airway ASA 1–2  Mallampati score 3–4 Age group 18–55 years old | Fentanyl + ketamine: fentanyl 2 µg.kg^-1^ bolus and ketamine 0.25 µg.kg^-1^ bolus  Dexmedetomidine: 1 µg.kg^-1^ over 10 min followed by infusion at a rate of 0.2–0.7 µg.kg^-1^hr^-1^ | Not specified | Not specified | Lidocaine  Xylometazoline |
| Xu et al 2016 [51] | Dexmedetomidine (34)  Remifentanil (34) | Patients scheduled for elective or urgent cervical spine surgery with cervical trauma or severe cervical spondylosis under general anaesthesia ASA 1–3 Mallampati score not specified  Age group 18–70 years old | Dexmedetomidine: 1 µg.kg^-1^ over 10 min followed by infusion at a rate of 0.2–0.7 µg.kg^-1^hr^-1^  Remifentanil: TCI 2.5 ng.ml^-1^ for 10 min followed by 3 ng.ml^-1^ | Separate | Consultant anaesthetists | Lidocaine |
| Yadav et al 2020 [52] | Midazolam + fentanyl (15)  Midazolam + dexmedetomidine (15) | Patients scheduled for elective surgery under general anaesthesia with anticipated difficult airway ASA 1–2  Mallampati score not specified  Age group 18 years or older | Midazolam + fentanyl: midazolam 0.02 mg.kg^−1^ and fentanyl 2 µg.kg^-1^ over 10 min  Midazolam + dexmedetomidine: midazolam 0.02 mg.kg^−1^ and dexmedetomidine 1 µg.kg^-1^ over 10 min | Same | Experienced anaesthetists | Lidocaine  Xylometazoline |
| Yousuf et al 2017 [53] | Midazolam + fentanyl (30)  Dexmedetomidine (30) | Patients scheduled for elective surgery under general anaesthesia with anticipated difficult airway  ASA 1–2  Mallampati score not specified  Age group 18–60 years old | Midazolam + fentanyl: midazolam 0.02 mg.kg^-1^ followed by fentanyl 2 µg.kg^-1^ over 10 min  Dexmedetomidine: 1 µg.kg^-1^ over 10 min | Not specified | Not specified | Lidocaine |
| Zhang et al 2012 [54] | Remifentanil (18)  Propofol (18) | Patients scheduled for elective surgery under general anaesthesia with anticipated difficult airway  ASA 1–2 Mallampati 1–4 Age group 18–65 years old | Remifentanil: TCI 2.5–3.5 ng.ml^-1^  Propofol: TCI 1.5–2.5 µg.ml^-1^ | Separate | Consultant anaesthetists | Lidocaine  Oxymetazoline |

TCI, target controlled infusion

**Table S2** Network league table for all the interventions in regard to overall awake tracheal intubation success rate, time to tracheal intubation and incidence of arterial oxygen desaturation. Estimates are presented as odds ratios or mean differences with 95% confidence interval in parentheses. In regard to overall awake tracheal intubation success rate, odds ratios above 1 favour the column intervention and odds ratios below 1 favour the row intervention. Interventions in bold are significantly different since the 95% confidence interval does not include 1. With respect to time to awake tracheal intubation, mean differences below 0 favour the column intervention and mean differences above 0 favour the row intervention. Interventions in bold are significantly different since the 95% confidence interval does not include 0. In regard to incidence of arterial oxygen desaturation, odds ratios below 1 favour the column intervention and odds ratios above 1 favour the row intervention. Interventions in bold are significantly different since the 95% confidence interval does not include 1.

***Overall awake tracheal intubation success rate (%)***

| **Alfentanil** |  |  |  |  |  |  |  |  |  |  |  |  |  |  |  |  |  |  |  |  |
| --- | --- | --- | --- | --- | --- | --- | --- | --- | --- | --- | --- | --- | --- | --- | --- | --- | --- | --- | --- | --- |
| 1.00 (0.02 to 51.87) | **Dexmedetomidine** |  |  |  |  |  |  |  |  |  |  |  |  |  |  |  |  |  |  |  |
| 1.00 (0.00 to 266.12) | 1.00 (0.02 to 51.84) | **Dexmedetomidine + Fentanyl** |  |  |  |  |  |  |  |  |  |  |  |  |  |  |  |  |  |  |
| 1.03 (0.01 to 166.03) | 1.03 (0.04 to 25.23) | 1.03 (0.01 to 166.02) | **Dexmedetomidine + Ketamine** |  |  |  |  |  |  |  |  |  |  |  |  |  |  |  |  |  |
| 1.06 (0.01 to 183.53) | 1.06 (0.04 to 29.17) | 1.06 (0.01 to 183.53) | 1.02 (0.06 to 17.23) | **Dexmedetomidine + Propofol** |  |  |  |  |  |  |  |  |  |  |  |  |  |  |  |  |
| 1.00 (0.01 to 95.92) | 1.00 (0.10 to 9.85) | 1.00 (0.01 to 95.88) | 0.97 (0.02 to 49.21) | 0.95 (0.02 to 53.24) | **Fentanyl** |  |  |  |  |  |  |  |  |  |  |  |  |  |  |  |
| 1.00 (0.00 to 266.82) | 1.00 (0.02 to 52.04) | 1.00 (0.00 to 266.74) | 0.97 (0.01 to 155.80) | 0.95 (0.01 to 164.80) | 1.00 (0.01 to 96.19) | **Fentanyl + Ketamine** |  |  |  |  |  |  |  |  |  |  |  |  |  |  |
| 1.21 (0.01 to 160.86) | 1.21 (0.07 to 21.70) | 1.21 (0.01 to 160.81) | 1.17 (0.02 to 85.74) | 1.14 (0.01 to 91.33) | 1.21 (0.03 to 48.09) | 1.21 (0.01 to 161.29) | **Fentanyl + Propofol** |  |  |  |  |  |  |  |  |  |  |  |  |  |
| 1.05 (0.00 to 299.07) | 1.05 (0.02 to 60.02) | 1.05 (0.00 to 299.06) | 1.01 (0.04 to 23.12) | 0.99 (0.04 to 22.56) | 1.05 (0.01 to 109.52) | 1.05 (0.00 to 299.83) | 0.87 (0.01 to 123.99) | **Ketamine + Propofol** |  |  |  |  |  |  |  |  |  |  |  |  |
| 1.19 (0.01 to 123.76) | 1.19 (0.10 to 13.77) | 1.19 (0.01 to 123.74) | 1.15 (0.02 to 63.99) | 1.12 (0.02 to 68.82) | 1.19 (0.04 to 33.91) | 1.19 (0.01 to 124.13) | 0.98 (0.02 to 40.67) | 1.13 (0.01 to 128.33) | **Midazolam** |  |  |  |  |  |  |  |  |  |  |  |
| 1.18 (0.00 to 370.32) | 1.18 (0.02 to 77.11) | 1.18 (0.00 to 370.27) | 1.14 (0.01 to 218.30) | 1.11 (0.01 to 229.34) | 1.18 (0.01 to 138.36) | 1.18 (0.00 to 371.21) | 0.98 (0.01 to 146.01) | 1.13 (0.00 to 377.19) | 0.99 (0.03 to 38.08) | **Midazolam + Clonidine** |  |  |  |  |  |  |  |  |  |  |
| 1.17 (0.01 to 156.02) | 1.17 (0.06 to 21.10) | 1.17 (0.01 to 155.99) | 1.13 (0.02 to 83.22) | 1.10 (0.01 to 88.64) | 1.17 (0.03 to 46.70) | 1.17 (0.01 to 156.46) | 0.97 (0.02 to 50.97) | 1.12 (0.01 to 160.14) | 0.98 (0.08 to 11.43) | 0.99 (0.03 to 38.08) | **Midazolam + Dexmedetomidine** |  |  |  |  |  |  |  |  |  |
| 1.45 (0.02 to 96.70) | 1.45 (0.35 to 6.05) | 1.45 (0.02 to 96.66) | 1.40 (0.04 to 45.25) | 1.37 (0.04 to 48.74) | 1.45 (0.10 to 21.52) | 1.45 (0.02 to 97.00) | 1.20 (0.07 to 21.64) | 1.39 (0.02 to 98.94) | 1.22 (0.09 to 17.37) | 1.23 (0.02 to 86.11) | 1.25 (0.07 to 22.59) | **Midazolam + Fentanyl** |  |  |  |  |  |  |  |  |
| 1.77 (0.02 to 181.56) | 1.77 (0.16 to 19.90) | 1.77 (0.02 to 181.51) | 1.71 (0.03 to 92.57) | 1.67 (0.03 to 98.87) | 1.77 (0.06 to 49.43) | 1.77 (0.02 to 182.08) | 1.47 (0.04 to 51.71) | 1.69 (0.02 to 185.88) | 1.49 (0.05 to 41.83) | 1.50 (0.01 to 167.83) | 1.52 (0.04 to 53.86) | 1.22 (0.13 to 11.07) | **Midazolam + Propofol** |  |  |  |  |  |  |  |
| 1.28 (0.00 to 582.20) | 1.28 (0.01 to 137.33) | 1.28 (0.00 to 582.07) | 1.24 (0.00 to 352.27) | 1.21 (0.00 to 366.81) | 1.28 (0.01 to 233.25) | 1.28 (0.00 to 583.46) | 1.06 (0.01 to 225.08) | 1.23 (0.00 to 586.40) | 1.08 (0.01 to 197.11) | 1.09 (0.00 to 527.48) | 1.10 (0.01 to 233.89) | 0.88 (0.01 to 84.98) | 0.72 (0.01 to 39.52) | **Midazolam + Remifentanil** |  |  |  |  |  |  |
| 1.17 (0.00 to 632.52) | 1.17 (0.01 to 157.25) | 1.17 (0.00 to 632.46) | 1.13 (0.00 to 389.77) | 1.10 (0.00 to 406.05) | 1.17 (0.01 to 261.16) | 1.17 (0.00 to 633.92) | 0.97 (0.00 to 262.11) | 1.12 (0.00 to 639.27) | 0.98 (0.01 to 103.50) | 0.99 (0.00 to 215.81) | 1.00 (0.02 to 52.36) | 0.80 (0.01 to 108.40) | 0.66 (0.00 to 136.05) | 0.91 (0.00 to 713.06) | **Midazolam + Sufentanil** |  |  |  |  |  |
| 0.74 (0.01 to 46.38) | 0.74 (0.21 to 2.59) | 0.74 (0.01 to 46.36) | 0.71 (0.02 to 22.04) | 0.70 (0.02 to 24.18) | 0.74 (0.05 to 10.01) | 0.74 (0.01 to 46.52) | 0.61 (0.03 to 14.26) | 0.70 (0.01 to 48.89) | 0.62 (0.04 to 9.75) | 0.63 (0.01 to 49.33) | 0.63 (0.03 to 14.82) | 0.51 (0.08 to 3.39) | 0.42 (0.03 to 6.36) | 0.57 (0.00 to 72.68) | 0.63 (0.00 to 99.70) | **Placebo** |  |  |  |  |
| 1.09 (0.01 to 82.93) | 1.09 (0.19 to 6.45) | 1.09 (0.01 to 82.91) | 1.06 (0.04 to 29.12) | 1.03 (0.04 to 25.32) | 1.09 (0.06 to 19.78) | 1.09 (0.01 to 83.19) | 0.91 (0.03 to 25.96) | 1.05 (0.02 to 60.06) | 0.92 (0.05 to 18.62) | 0.93 (0.01 to 85.66) | 0.94 (0.03 to 26.99) | 0.75 (0.09 to 6.57) | 0.62 (0.03 to 11.68) | 0.85 (0.01 to 122.10) | 0.94 (0.01 to 168.58) | 1.49 (0.17 to 13.10) | **Propofol** |  |  |  |
| 0.78 (0.01 to 51.98) | 0.78 (0.19 to 3.24) | 0.78 (0.01 to 51.96) | 0.76 (0.03 to 21.62) | 0.74 (0.03 to 21.62) | 0.78 (0.05 to 11.56) | 0.78 (0.01 to 52.14) | 0.65 (0.03 to 15.30) | 0.75 (0.01 to 46.85) | 0.66 (0.04 to 10.84) | 0.67 (0.01 to 53.40) | 0.67 (0.03 to 15.92) | 0.54 (0.09 to 3.31) | 0.44 (0.03 to 6.61) | 0.61 (0.00 to 76.31) | 0.67 (0.00 to 106.68) | 1.06 (0.16 to 7.10) | 0.72 (0.15 to 3.53) | **Remifentanil** |  |  |
| 0.78 (0.00 to 254.64) | 0.78 (0.01 to 53.67) | 0.78 (0.00 to 254.58) | 0.76 (0.00 to 137.98) | 0.74 (0.00 to 136.90) | 0.78 (0.01 to 95.78) | 0.78 (0.00 to 255.22) | 0.65 (0.00 to 104.74) | 0.75 (0.00 to 233.34) | 0.66 (0.01 to 85.79) | 0.67 (0.00 to 248.72) | 0.67 (0.00 to 108.74) | 0.54 (0.01 to 42.86) | 0.44 (0.00 to 54.49) | 0.61 (0.00 to 319.08) | 0.67 (0.00 to 422.96) | 1.06 (0.01 to 87.69) | 0.72 (0.01 to 52.24) | 1.00 (0.02 to 53.66) | **Remifentanil + Propofol** |  |
| 1.00 (0.00 to 269.77) | 1.00 (0.02 to 52.85) | 1.00 (0.00 to 269.68) | 0.97 (0.01 to 157.69) | 0.95 (0.01 to 166.77) | 1.00 (0.01 to 97.49) | 1.00 (0.00 to 270.39) | 0.83 (0.01 to 112.29) | 0.96 (0.00 to 277.43) | 0.84 (0.01 to 89.37) | 0.85 (0.00 to 271.26) | 0.86 (0.01 to 116.55) | 0.69 (0.01 to 46.66) | 0.57 (0.01 to 58.96) | 0.78 (0.00 to 359.17) | 0.86 (0.00 to 470.91) | 1.36 (0.02 to 87.35) | 0.92 (0.01 to 70.67) | 1.28 (0.02 to 86.46) | 1.28 (0.00 to 421.52) | **Sufentanil** |

***Time to tracheal intubation (s)***

| **Alfentanil** |  |  |  |  |  |  |  |  |  |  |  |  |  |  |  |  |  |  |  |
| --- | --- | --- | --- | --- | --- | --- | --- | --- | --- | --- | --- | --- | --- | --- | --- | --- | --- | --- | --- |
| -12.18 (-91.64 to 67.28) | **Dexmedetomidine** |  |  |  |  |  |  |  |  |  |  |  |  |  |  |  |  |  |  |
| **458.56 (250.12 to 667.00)** | **470.74 (272.30 to 669.18)** | **Dexmedetomidine + Ketamine** |  |  |  |  |  |  |  |  |  |  |  |  |  |  |  |  |  |
| -16.73 (-176.07 to 142.62) | -4.55 (-149.20 to 140.11) | **-475.28 (-617.21 to -333.36)** | **Dexmedetomidine + Propofol** |  |  |  |  |  |  |  |  |  |  |  |  |  |  |  |  |
| -97.02 (-207.93 to 13.89) | **-84.84 (-162.93 to -6.76)** | **-555.58 (-768.18 to -342.98)** | -80.30 (-244.31 to 83.71) | **Fentanyl** |  |  |  |  |  |  |  |  |  |  |  |  |  |  |  |
| -13.23 (-138.90 to 112.44) | -1.05 (-99.22 to 97.11) | **-471.79 (-693.14 to -250.44)** | 3.50 (-171.27 to 178.26) | 83.79 (-41.64 to 209.23) | **Fentanyl + Ketamine** |  |  |  |  |  |  |  |  |  |  |  |  |  |  |
| -0.03 (-133.92 to 133.86) | 12.15 (-96.34 to 120.64) | **-458.59 (-684.71 to -232.47)** | 16.70 (-164.07 to 197.46) | 96.99 (-36.68 to 230.66) | 13.20 (-133.10 to 159.50) | **Fentanyl + Propofol** |  |  |  |  |  |  |  |  |  |  |  |  |  |
| **459.77 (274.37 to 645.16)** | **471.94 (298.08 to 645.81)** | 1.21 (-97.00 to 99.42) | **476.49 (373.38 to 579.60)** | **556.79 (366.78 to 746.79)** | **473.00 (273.39 to 672.60)** | **459.80 (254.91 to 664.68)** | **Ketamine + Propofol** |  |  |  |  |  |  |  |  |  |  |  |  |
| **217.98 (29.44 to 406.52)** | **230.16 (53.29 to 407.02)** | -240.58 (-504.72 to 23.55) | **234.70 (7.36 to 462.04)** | **315.00 (121.96 to 508.03)** | **231.21 (28.98 to 433.43)** | **218.01 (10.57 to 425.44)** | -241.79 (-488.23 to 4.66) | **Magnesium sulphate** |  |  |  |  |  |  |  |  |  |  |  |
| -96.47 (-255.25 to 62.32) | -84.29 (-228.50 to 59.92) | **-555.03 (-798.83 to -311.23)** | -79.74 (-282.91 to 123.42) | 0.55 (-163.17 to 164.27) | -83.24 (-257.63 to 91.15) | -96.44 (-276.85 to 83.97) | **-556.23 (-780.69 to -331.78)** | **-314.45 (-419.73 to -209.17)** | **Midazolam** |  |  |  |  |  |  |  |  |  |  |
| -41.51 (-202.63 to 119.62) | -29.33 (-175.72 to 117.07) | **-500.06 (-745.47 to -254.66)** | -24.78 (-229.68 to 180.12) | 55.52 (-110.20 to 221.23) | -28.28 (-204.48 to 147.92) | -41.48 (-223.63 to 140.68) | **-501.27 (-727.41 to -275.13)** | **-259.48 (-404.77 to -114.19)** | 54.96 (-45.85 to 155.77) | **Midazolam + Clonidine** |  |  |  |  |  |  |  |  |  |
| 20.22 (-107.55 to 147.99) | 32.40 (-74.89 to 139.69) | **-438.34 (-662.89 to -213.79)** | 36.95 (-142.31 to 216.20) | 117.24 (-15.26 to 249.75) | 33.45 (-111.91 to 178.81) | 20.25 (-132.27 to 172.77) | **-439.54 (-642.82 to -236.27)** | **-197.76 (-342.34 to -53.17)** | **116.69 (16.16 to 217.22)** | 61.73 (-40.67 to 164.12) | **Midazolam + Dexmedetomidine** |  |  |  |  |  |  |  |  |
| -22.09 (-114.59 to 70.40) | -9.91 (-63.02 to 43.20) | **-480.65 (-685.75 to -275.55)** | -5.37 (-159.15 to 148.42) | 74.93 (-19.45 to 169.31) | -8.86 (-120.44 to 102.72) | -22.06 (-142.82 to 98.70) | **-481.86 (-663.31 to -300.40)** | **-240.07 (-410.68 to -69.46)** | 74.38 (-61.84 to 210.60) | 19.41 (-118.90 to 157.73) | -42.31 (-137.64 to 53.02) | **Midazolam + Fentanyl** |  |  |  |  |  |  |  |
| -22.79 (-140.07 to 94.48) | -10.62 (-100.27 to 79.04) | **-481.35 (-698.90 to -263.81)** | -6.07 (-176.02 to 163.88) | 74.23 (-44.65 to 193.11) | -9.56 (-142.48 to 123.35) | -22.76 (-163.48 to 117.95) | **-482.56 (-677.94 to -287.18)** | **-240.77 (-432.83 to -48.71)** | 73.67 (-88.72 to 236.07) | 18.71 (-145.54 to 182.96) | -43.01 (-173.42 to 87.39) | -0.70 (-90.94 to 89.54) | **Midazolam + Propofol** |  |  |  |  |  |  |
| 12.49 (-148.05 to 173.02) | 24.66 (-117.48 to 166.81) | **-446.07 (-689.96 to -202.19)** | 29.21 (-173.36 to 231.78) | 109.51 (-52.67 to 271.68) | 25.72 (-147.00 to 198.44) | 12.52 (-166.28 to 191.31) | **-447.28 (-671.62 to -222.94)** | -205.49 (-427.00 to 16.01) | 108.95 (-87.39 to 305.30) | 53.99 (-143.89 to 251.87) | -7.74 (-178.58 to 163.11) | 34.58 (-108.03 to 177.19) | 35.28 (-75.88 to 146.44) | **Midazolam + Remifentanil** |  |  |  |  |  |
| 43.97 (-121.61 to 209.55) | 56.15 (-94.63 to 206.94) | **-414.59 (-662.81 to -166.36)** | 60.70 (-147.47 to 268.87) | 140.99 (-28.66 to 310.64) | 57.20 (-122.67 to 237.07) | 44.00 (-141.71 to 229.71) | **-415.79 (-644.96 to -186.63)** | -174.01 (-353.92 to 5.91) | 140.44 (-6.45 to 287.34) | 85.48 (-62.72 to 233.67) | 23.75 (-83.43 to 130.94) | 66.06 (-76.66 to 208.79) | 66.77 (-101.32 to 234.86) | 31.49 (-169.60 to 232.57) | **Midazolam + Sufentanil** |  |  |  |  |
| **-567.91 (-758.66 to -377.17)** | **-555.73 (-734.93 to -376.54)** | **-1026.47 (-1292.17 to -760.77)** | **-551.19 (-780.35 to -322.03)** | **-470.89 (-666.06 to -275.72)** | **-554.68 (-758.95 to -350.42)** | **-567.88 (-777.31 to -358.46)** | **-1027.68 (-1275.80 to -779.56)** | **-785.89 (-890.09 to -681.69)** | **-471.44 (-580.51 to -362.38)** | **-526.41 (-674.47 to -378.34)** | **-588.13 (-735.53 to -440.74)** | **-545.82 (-718.85 to -372.80)** | **-545.12 (-739.33 to -350.91)** | **-580.40 (-803.77 to -357.03)** | **-611.89 (-794.07 to -429.71)** | **Placebo** |  |  |  |
| -14.39 (-119.33 to 90.55) | -2.21 (-74.78 to 70.36) | **-472.95 (-661.50 to -284.39)** | 2.34 (-127.04 to 131.72) | 82.63 (-23.81 to 189.07) | -1.16 (-123.21 to 120.89) | -14.36 (-144.86 to 116.14) | **-474.15 (-636.41 to -311.90)** | **-232.37 (-423.14 to -41.59)** | 82.08 (-78.96 to 243.12) | 27.12 (-135.94 to 190.17) | -34.61 (-163.79 to 94.57) | 7.70 (-82.06 to 97.47) | 8.41 (-106.83 to 123.64) | -26.87 (-186.38 to 132.63) | -58.36 (-225.41 to 108.69) | **553.52 (360.58 to 746.46)** | **Propofol** |  |  |
| 48.07 (-43.55 to 139.69) | **60.25 (11.92 to 108.57)** | **-410.49 (-614.47 to -206.52)** | 64.79 (-87.51 to 217.10) | **145.09 (53.33 to 236.84)** | 61.30 (-48.11 to 170.70) | 48.10 (-70.66 to 166.85) | **-411.70 (-591.89 to -231.51)** | -169.91 (-350.52 to 10.70) | 144.54 (-4.22 to 293.30) | 89.57 (-61.28 to 240.43) | 27.85 (-85.38 to 141.08) | **70.16 (6.34 to 133.98)** | 70.86 (-28.38 to 170.11) | 35.58 (-112.81 to 183.98) | 4.10 (-151.00 to 159.19) | **615.98 (433.09 to 798.87)** | 62.46 (-24.62 to 149.54) | **Remifentanil** |  |
| -12.03 (-262.92 to 238.86) | 0.15 (-238.15 to 238.45) | **-470.59 (-780.66 to -160.51)** | 4.70 (-274.04 to 283.43) | 84.99 (-165.78 to 335.76) | 1.20 (-256.53 to 258.93) | -12.00 (-273.83 to 249.83) | **-471.80 (-766.75 to -176.84)** | -230.01 (-526.74 to 66.72) | 84.44 (-194.07 to 362.94) | 29.48 (-250.17 to 309.12) | -32.25 (-293.56 to 229.06) | 10.06 (-234.07 to 254.20) | 10.76 (-243.83 to 265.36) | -24.52 (-301.98 to 252.95) | -56.00 (-337.97 to 225.97) | **555.88 (257.76 to 854.01)** | 2.36 (-246.74 to 251.46) | -60.10 (-303.25 to 183.05) | **Sufentanil** |

***Incidence of arterial oxygen desaturation (%)***

| **Alfentanil** |  |  |  |  |  |  |  |  |  |  |  |  |  |  |  |  |  |  |  |
| --- | --- | --- | --- | --- | --- | --- | --- | --- | --- | --- | --- | --- | --- | --- | --- | --- | --- | --- | --- |
| 1.00 (0.01 to 67.62) | **Dexmedetomidine** |  |  |  |  |  |  |  |  |  |  |  |  |  |  |  |  |  |  |
| 0.59 (0.00 to 138.70) | 0.59 (0.02 to 19.01) | **Dexmedetomidine + Ketamine** |  |  |  |  |  |  |  |  |  |  |  |  |  |  |  |  |  |
| 0.35 (0.00 to 87.08) | 0.35 (0.01 to 12.34) | 0.59 (0.02 to 18.97) | **Dexmedetomidine + Propofol** |  |  |  |  |  |  |  |  |  |  |  |  |  |  |  |  |
| 0.20 (0.00 to 16.62) | **0.20 (0.05 to 0.80)** | 0.33 (0.01 to 14.07) | 0.56 (0.01 to 25.98) | **Fentanyl** |  |  |  |  |  |  |  |  |  |  |  |  |  |  |  |
| 1.00 (0.00 to 388.16) | 1.00 (0.01 to 67.80) | 1.70 (0.01 to 399.95) | 2.87 (0.01 to 718.36) | 5.11 (0.06 to 435.87) | **Fentanyl + Ketamine** |  |  |  |  |  |  |  |  |  |  |  |  |  |  |
| 0.32 (0.00 to 79.72) | 0.32 (0.01 to 11.33) | 0.54 (0.00 to 78.66) | 0.91 (0.01 to 142.10) | 1.62 (0.03 to 75.70) | 0.32 (0.00 to 79.87) | **Fentanyl + Propofol** |  |  |  |  |  |  |  |  |  |  |  |  |  |
| 0.35 (0.00 to 360.87) | 0.35 (0.00 to 86.79) | 0.59 (0.00 to 138.17) | 1.00 (0.01 to 67.30) | 1.78 (0.01 to 530.02) | 0.35 (0.00 to 361.49) | 1.10 (0.00 to 788.23) | **Ketamine + Propofol** |  |  |  |  |  |  |  |  |  |  |  |  |
| 47.60 (0.12 to 19626.52) | 47.58 (0.64 to 3514.73) | 80.71 (0.32 to 20336.64) | 136.51 (0.51 to 36501.37) | **243.38 (2.63 to 22497.39)** | 47.58 (0.12 to 19663.35) | 150.07 (0.56 to 40364.20) | 136.51 (0.12 to 149179.23) | **Magnesium sulphate** |  |  |  |  |  |  |  |  |  |  |  |
| 1.63 (0.01 to 305.14) | 1.63 (0.07 to 36.17) | 2.77 (0.03 to 290.91) | 4.69 (0.04 to 527.87) | 8.36 (0.28 to 251.46) | 1.63 (0.01 to 305.79) | 5.15 (0.05 to 584.49) | 4.69 (0.01 to 2623.05) | **0.03 (0.00 to 0.86)** | **Midazolam** |  |  |  |  |  |  |  |  |  |  |
| 0.84 (0.00 to 175.61) | 0.84 (0.03 to 22.45) | 1.42 (0.01 to 169.27) | 2.41 (0.02 to 305.70) | 4.29 (0.12 to 151.51) | 0.84 (0.00 to 176.00) | 2.64 (0.02 to 340.40) | 2.41 (0.00 to 1473.98) | 0.02 (0.00 to 3.96) | 0.51 (0.01 to 47.07) | **Midazolam + Dexmedetomidine** |  |  |  |  |  |  |  |  |  |
| 0.39 (0.00 to 34.35) | 0.39 (0.09 to 1.78) | 0.66 (0.01 to 29.16) | 1.12 (0.02 to 53.56) | 1.99 (0.26 to 15.51) | 0.39 (0.00 to 34.44) | 1.23 (0.03 to 59.83) | 1.12 (0.00 to 339.88) | **0.01 (0.00 to 0.78)** | 0.24 (0.01 to 7.53) | 0.46 (0.03 to 8.56) | **Midazolam + Fentanyl** |  |  |  |  |  |  |  |  |
| 0.20 (0.00 to 34.69) | 0.20 (0.01 to 3.90) | 0.34 (0.00 to 32.72) | 0.57 (0.01 to 59.36) | 1.02 (0.04 to 27.23) | 0.20 (0.00 to 34.76) | 0.63 (0.01 to 65.93) | 0.57 (0.00 to 301.38) | **0.00 (0.00 to 0.78)** | 0.12 (0.00 to 8.96) | 0.24 (0.00 to 15.31) | 0.51 (0.03 to 10.03) | **Midazolam + Propofol** |  |  |  |  |  |  |  |
| 0.28 (0.00 to 221.87) | 0.28 (0.00 to 49.80) | 0.47 (0.00 to 242.07) | 0.79 (0.00 to 431.06) | 1.41 (0.01 to 305.99) | 0.28 (0.00 to 222.23) | 0.87 (0.00 to 477.46) | 0.79 (0.00 to 1545.41) | 0.01 (0.00 to 4.93) | 0.17 (0.00 to 71.59) | 0.33 (0.00 to 127.19) | 0.71 (0.00 to 127.91) | 1.38 (0.02 to 97.91) | **Midazolam + Remifentanil** |  |  |  |  |  |  |
| 0.53 (0.00 to 186.38) | 0.53 (0.01 to 31.10) | 0.91 (0.00 to 189.65) | 1.53 (0.01 to 340.18) | 2.73 (0.04 to 199.72) | 0.53 (0.00 to 186.75) | 1.69 (0.01 to 378.29) | 1.53 (0.00 to 1444.96) | 0.01 (0.00 to 4.18) | 0.33 (0.00 to 54.28) | 0.64 (0.06 to 6.95) | 1.37 (0.03 to 59.47) | 2.68 (0.02 to 324.88) | 1.94 (0.00 to 1187.35) | **Midazolam + Sufentanil** |  |  |  |  |  |
| 0.20 (0.00 to 89.33) | 0.20 (0.00 to 16.64) | 0.33 (0.00 to 93.42) | 0.56 (0.00 to 167.54) | 1.00 (0.01 to 67.74) | 0.20 (0.00 to 89.49) | 0.62 (0.00 to 185.12) | 0.56 (0.00 to 669.84) | 0.00 (0.00 to 2.00) | 0.12 (0.00 to 26.98) | 0.23 (0.00 to 58.25) | 0.50 (0.00 to 54.60) | 0.98 (0.00 to 204.57) | 0.71 (0.00 to 658.12) | 0.37 (0.00 to 149.77) | **Nalbuphine** |  |  |  |  |
| 0.61 (0.00 to 114.23) | 0.61 (0.03 to 13.54) | 1.04 (0.01 to 108.92) | 1.75 (0.02 to 197.69) | 3.13 (0.10 to 93.72) | 0.61 (0.00 to 114.47) | 1.93 (0.02 to 218.81) | 1.75 (0.00 to 982.21) | **0.01 (0.00 to 0.32)** | 0.37 (0.07 to 1.99) | 0.73 (0.01 to 66.62) | 1.57 (0.05 to 49.38) | 3.06 (0.04 to 223.70) | 2.22 (0.01 to 937.99) | 1.14 (0.01 to 189.02) | 3.13 (0.01 to 703.64) | **Placebo** |  |  |  |
| 0.21 (0.00 to 17.31) | **0.21 (0.05 to 0.82)** | 0.35 (0.01 to 12.28) | 0.59 (0.02 to 19.03) | 1.05 (0.15 to 7.58) | 0.21 (0.00 to 17.35) | 0.65 (0.01 to 29.95) | 0.59 (0.00 to 138.22) | **0.00 (0.00 to 0.40)** | 0.13 (0.00 to 3.73) | 0.24 (0.01 to 8.56) | 0.53 (0.07 to 4.03) | 1.03 (0.04 to 27.07) | 0.74 (0.00 to 160.11) | 0.38 (0.01 to 27.78) | 1.05 (0.01 to 110.40) | 0.34 (0.01 to 9.98) | **Propofol** |  |  |
| 0.43 (0.01 to 32.46) | 0.43 (0.15 to 1.18) | 0.72 (0.02 to 25.92) | 1.22 (0.03 to 46.29) | 2.17 (0.38 to 12.33) | 0.43 (0.01 to 32.55) | 1.34 (0.03 to 55.26) | 1.22 (0.00 to 317.68) | **0.01 (0.00 to 0.74)** | 0.26 (0.01 to 6.78) | 0.51 (0.02 to 15.22) | 1.09 (0.19 to 6.31) | 2.13 (0.09 to 48.20) | 1.54 (0.01 to 303.03) | 0.80 (0.01 to 50.78) | 2.17 (0.02 to 207.62) | 0.70 (0.03 to 18.12) | 2.07 (0.48 to 8.98) | **Remifentanil** |  |
| 1.33 (0.01 to 147.45) | 1.33 (0.16 to 10.83) | 2.26 (0.04 to 130.58) | 3.83 (0.06 to 239.41) | 6.82 (0.55 to 85.04) | 1.33 (0.01 to 147.79) | 4.21 (0.07 to 265.29) | 3.83 (0.01 to 1398.54) | 0.03 (0.00 to 3.35) | 0.82 (0.02 to 34.32) | 1.59 (0.03 to 78.39) | 3.42 (0.26 to 45.58) | 6.67 (0.18 to 253.16) | 4.83 (0.02 to 1308.88) | 2.49 (0.03 to 241.10) | 6.82 (0.05 to 927.91) | 2.18 (0.05 to 91.70) | 6.50 (0.53 to 80.16) | 3.14 (0.31 to 32.20) | **Sufentanil** |

**Table S3** Full trial details and conclusions from the network meta-analysis.

| **Outcome** | **Trials** | **Participants** | **Interventions** | **Direct comparisons** | **Indirect comparisons** | **Mean (SD) across all trials** | **Conclusions** | **Quality of evidence** | **Comments** |
| --- | --- | --- | --- | --- | --- | --- | --- | --- | --- |
| Overall awake tracheal intubation success rate (%) [14,15,28–34,36–38,17,39–41,43–49,19,50–58,62,20,22,23,25–27] | 40 | 2246 | 21 | 28 | 182 | 99 (2.2) | No differences between interventions | Low (⊕⊕) | No local or global inconsistency  Downgraded for serious limitations and imprecision |
| Time to tracheal intubation (s) [16,17,31–33,37,39,40,43,45,47,49,19,50–52,56,20–26] | 24 | 1745 | 20 | 23 | 167 | 237 (319) | Placebo inferior to all other interventions  Dexmedetomidine superior to fentanyl  Dexmedetomidine + ketamine superior to alfentanil, dexmedetomidine, dexmedetomidine + propofol, fentanyl, fentanyl + ketamine, fentanyl + propofol, midazolam, midazolam + clonidine, midazolam + dexmedetomidine, midazolam + fentanyl, midazolam + propofol, midazolam + remifentanil, midazolam + sufentanil, placebo, propofol, remifentanil and sufentanil  Ketamine + propofol superior to alfentanil, dexmedetomidine, dexmedetomidine + propofol, fentanyl, fentanyl + ketamine, fentanyl + propofol, midazolam, midazolam + clonidine, midazolam + dexmedetomidine, midazolam + fentanyl, midazolam + propofol, midazolam + remifentanil, midazolam + sufentanil, placebo, propofol, remifentanil and sufentanil  Magnesium sulphate superior to alfentanil, dexmedetomidine, dexmedetomidine + propofol, fentanyl, fentanyl + ketamine, fentanyl + propofol, midazolam, midazolam + clonidine, midazolam + dexmedetomidine, midazolam + fentanyl, midazolam + propofol, placebo and propofol  Midazolam + dexmedetomidine superior to midazolam  Remifentanil superior to dexmedetomidine, fentanyl, midazolam + fentanyl and placebo | Low (⊕⊕) | No local or global inconsistency  Downgraded for serious limitations and imprecision |
| Incidence of arterial oxygen desaturation (%) [16,17,31–34,36,38–41,43,19,45–47,49–54,58,20,60,62,21,23,25,27,29,30] | 32 | 1813 | 20 | 25 | 165 | 10 (18.5) | Dexmedetomidine superior to fentanyl and propofol  Magnesium sulphate superior to fentanyl, midazolam, midazolam + fentanyl, midazolam + propofol, placebo, propofol and remifentanil | Low (⊕⊕) | No local or global inconsistency  Downgraded for serious limitations and imprecision |
| Need for rescue sedation (%) [24,29,32,33,43,47,48,54,61] | 9 | 578 | 7 | 7 | 14 | 26 (31.9) | Dexmedetomidine and remifentanil superior to placebo | Low (⊕⊕) | No local or global inconsistency  Downgraded for serious imprecision and publication bias |
| Time to conduct fibrescopy(s) [24,25,33,40,43,50,56] | 7 | 351 | 7 | 7 | 14 | 132 (56.4) | Data not available for placebo  No differences between interventions | Low (⊕⊕) | No local or global inconsistency  Downgraded for serious limitations and imprecision |
| Incidence of adverse cardiovascular events [14,16,37,38,41,45–51,17,52,53,55,58,62,20,23,25,29–31,34] | 25 | 1323 | 18 | 20 | 133 | 21 (25.8) | Alfentanil superior to dexmedetomidine, dexmedetomidine + fentanyl, fentanyl + propofol, midazolam, midazolam + fentanyl, placebo and sufentanil  Alfentanil, dexmedetomidine, dexmedetomidine + propofol, fentanyl, fentanyl + propofol, midazolam, midazolam + dexmedetomidine, midazolam + fentanyl, midazolam + sufentanil, placebo, propofol, remifentanil, remifentanil + propofol and sufentanil superior to dexmedetomidine + fentanyl  Propofol superior to fentanyl + propofol and midazolam | Low (⊕⊕) | No local or global inconsistency  Downgraded for serious limitations and imprecision |

**Table S4** Summary of the results from individual randomised controlled trials not included in the network meta-analysis.

| **Outcome** | **Trial** | **Results** |
| --- | --- | --- |
| Overall ATI success rate (%) | Chalam 2015 [59] | 100% with diazepam + dexmedetomidine and 98% with diazepam + propofol (no p value reported) |
|  | Eftekharian et al 2015 [18] | No difference between midazolam + fentanyl + ketamine, midazolam + fentanyl + propofol and midazolam + fentanyl + remifentanil (100% vs 100% vs 100%) |
| Time to tracheal intubation (s) | Eftekharian et al 2015 [18] | Midazolam + fentanyl + remifentanil superior to midazolam + fentanyl + ketamine and midazolam + fentanyl + propofol and midazolam + fentanyl + ketamine superior to midazolam + fentanyl + propofol (30.3 vs 46.4 vs 59.7 3 s; p < 0.001) |
|  | Randell et al 1990 [42] | Diazepam + alfentanil superior to diazepam (73 vs 111 s; p < 0.05) |
| Need for rescue sedation (%) | El Mourad et al 2019 [19] | Ketamine + propofol superior to dexmedetomidine + propofol (22.5% vs 47.5%; p = 0.035) |
|  | Randell et al 1990 [42] | Alfentanil + diazepam superior to diazepam (26.7% vs 86.7%; p < 0.001) |
| Incidence of adverse cardiovascular effects (%) | Rai et al 2008 [40] | No difference between midazolam + propofol and midazolam + remifentanil (0% vs 0%) |
